# Successional Dynamics and Seascape-Level Patterns of Microbial Communities on the Canopy-Forming Kelps *Nereocystis luetkeana* and *Macrocystis pyrifera*

**DOI:** 10.3389/fmicb.2019.00346

**Published:** 2019-02-26

**Authors:** Brooke L. Weigel, Catherine A. Pfister

**Affiliations:** ^1^Committee on Evolutionary Biology, University of Chicago, Chicago, IL, United States; ^2^Department of Ecology and Evolution, University of Chicago, Chicago, IL, United States

**Keywords:** kelp, microbiome, biogeography, community succession, holobiont, *Nereocystis*, *Macrocystis*, microbial symbiont

## Abstract

Canopy-forming kelps create underwater forests that are among the most productive marine ecosystems. On the Pacific coast of North America, two canopy-forming kelps with contrasting life histories co-occur; *Macrocystis pyrifera*, a perennial species, and *Nereocystis luetkeana*, an annual species. Kelp blade-associated microbes were sampled from 12 locations across a spatial gradient in Washington, United States, from the outer Pacific Coast to Puget Sound. Microbial communities were characterized using next-generation Illumina sequencing of 16S rRNA genes. At higher taxonomic levels (bacterial phylum and class), canopy-forming kelps hosted remarkably similar microbial communities, but at the amplicon sequence variant level, microbial communities on *M. pyrifera* and *N. luetkeana* were host-specific and distinct from free-living bacteria in the surrounding seawater. Microbial communities associated with blades of each kelp species displayed significant geographic variation. The microbiome of *N. luetkeana* changed along the spatial gradient and was significantly correlated to salinity, with outer Pacific coast sites enriched in *Bacteroidetes* (family *Saprospiraceae*) and *Gammaproteobacteria* (*Granulosicoccus* sp.), and southern Puget Sound sites enriched in *Alphaproteobacteria* (family *Hyphomonadaceae*). We also examined microbial community development and succession on meristematic and apical *N. luetkeana* blade tissues throughout the summer growing season on Tatoosh Island, WA. Across all dates, microbial communities were less diverse on younger, meristematic blade tissue compared to the older, apical tissues. In addition, phylogenetic relatedness among microbial taxa increased from meristematic to apical blade tissues, suggesting that the addition of microbial taxa to the community was a non-random process that selected for certain phylogenetic groups of microbes. Microbial communities on older, apical tissues displayed significant temporal variation throughout the summer and microbial taxa that were differentially abundant over time displayed clear patterns of community succession. Overall, we report that host species identity, geographic location, and blade tissue age shape the microbial communities on canopy-forming kelps.

## Introduction

Kelps (brown algae in the order Laminariales) are important habitat-forming foundational species in temperate coastal marine ecosystems worldwide (Krumhansl et al., 2016). Canopy-forming kelps create vast and highly productive underwater forests, fixing up to 1.3 kg C per m^2^ each year (Wheeler and Druehl, 1986). Macroalgae present an abundant surface for bacterial settlement, and they provide heterotrophic microbes with potential metabolic resources, including algal polysaccharides (Martin et al., 2015; Lin et al., 2018) and dissolved organic carbon (Abdullah and Fredriksen, 2004; Reed et al., 2015). As a result, kelps host an abundant epiphytic microbial community in their surface mucus layer (Bengtsson and Øvreås, 2010; Michelou et al., 2013; Lemay et al., 2018). Epiphytic microbial communities on macroalgae tend to be host species-specific (Lachnit et al., 2009; Bondoso et al., 2014; Aires et al., 2016), and they have a distinct composition from free-living microbial communities in the surrounding seawater (Staufenberger et al., 2008; Bengtsson et al., 2010; Michelou et al., 2013; Mancuso et al., 2016). The kelp microbiome can be an indicator of overall host health (Marzinelli et al., 2015) and exposure to environmental stressors (Minich et al., 2018). Microbial symbionts in coral reefs and the plant rhizosphere can contribute substantially to host health and may add adaptive capacity in changing environments (Correa and Baker, 2011; Philippot et al., 2013; Cunning et al., 2015). Recent research has shown that the microbiome of the canopy kelp *M. pyrifera* is susceptible to disruption by elevated temperatures (Minich et al., 2018), and there has been increasing concern about kelp forests declining in abundance in some regions of the world (Krumhansl et al., 2016; Pfister et al., 2018). Despite these concerns, we know little about the ecological and evolutionary processes that shape the microbiome of canopy-forming kelps.

Two species of canopy-forming kelps co-occur along the Pacific Coast of North America but have contrasting life histories: *Macrocystis pyrifera* is a perennial species that can persist for several years and *Nereocystis luetkeana* is an annual species. These two canopy-forming kelp species often coexist together in Washington kelp forests (Pfister et al., 2018), yet no studies have directly compared their microbial communities. One study found that epiphytic microbial communities among eight sympatric kelp species were structured by life history strategy (annual vs. perennial) rather than by host phylogeny (Lemay et al., 2018), indicating that the longevity of the thallus may affect microbial community assembly on kelps. Lemay et al. (2018) reported that the microbiome of *N. luetkeana* was similar to the microbial communities of other co-occurring annual kelps. However, all *N.* luetkeana samples in Lemay et al. (2018) were from the relatively young meristematic region at the base of the blade, which might have lower bacterial abundance and diversity than other regions of the kelp thallus (Staufenberger et al., 2008). The microbiome of *M. pyrifera* was first characterized in central California, with dominant bacterial phyla consisting of *Proteobacteria*, *Bacteroidetes*, and *Verrucomicrobia* (Michelou et al., 2013). In our study, we characterize the microbial communities of co-occurring *N. luetkeana* and *M. pyrifera*, using both spatial and temporal sampling to understand how host species identity, geographic location, and time shape the microbiome of these giant, ecologically important kelps.

First, we ask whether these kelps display geographic variation in blade-associated microbial communities. Examining the degree of similarity in microbial communities from one host species across a geographic scale can provide an understanding of how strongly the host, environmental variables, or geographic distance (i.e., dispersal limitation) structures the microbiome (Hanson et al., 2012; Pita et al., 2013). If microbial symbionts are environmentally acquired, which is likely on annual kelps, they may reflect the microbial composition of the surrounding seawater. The assembly of a host-specific microbiome may also relate to the strength of functional interactions between the host and its microbial associates (Burke et al., 2011; Fahimipour et al., 2017). For example, the leaf microbiome of eelgrass (*Zostera marina*) was spatially variable and similar to local seawater microbial communities, while the root microbiome contained a set of core taxa and remained distinct from sediment microbial communities across a global spatial scale (Fahimipour et al., 2017). Determining whether kelp-associated microbial communities change as the species pool in the surrounding seawater changes across large geographic distances is a first step toward understanding the stability and specificity of the interactions between canopy-forming kelps and their microbial associates. This study characterized the spatial dynamics of microbial communities associated with *N. luetkeana*, *M. pyrifera*, and seawater across a broad spatial gradient in Washington, encompassing significant environmental variation in temperature, salinity, and seawater nutrient concentrations.

In addition to the effects of host kelp species identity, environmental variables, and geographic distance, time may also be an important factor structuring kelp-associated microbial communities. For example, epiphytic biofilms on the kelp *Laminaria hyperborea* displayed temporal changes in cell density and bacterial community composition over 1 year. Bacterial cell densities were lowest (10^2^ cells cm^-2^) at the start of the kelp growing season, reaching a peak (10^7^ cells cm^-2^) after the end of the growing season (Bengtsson et al., 2010). The epiphytic microbiome of the brown algae *Cystoseira compressa* displayed a successional pattern, with a gradual increase in the number of bacterial taxa throughout the growing season (Mancuso et al., 2016). Another study examined the microbiome of *M. pyrifera*, finding temporal variation in the relative abundances of dominant bacterial phyla (*Proteobacteria*, *Bacteroidetes*, *Verrucomicrobia*) between kelp sampled in March and May (Michelou et al., 2013). While it is clear that microbial communities on kelp undergo temporal or seasonal changes, studies with high-resolution sequencing and greater replication are necessary to characterize the temporal dynamics of the kelp microbiome.

Canopy kelp blades grow outward from the float at extraordinarily high rates, expanding linearly 1–2 cm each day (Brown et al., 1997). This creates tissues of varying age along the length of the blade, which can be used to examine how kelp thallus age affects microbial community development. The first studies to recognize this cultured and counted colonies of bacteria from basal meristem, middle, and tip regions of *Laminaria* spp. fronds, finding that bacterial densities were highest at the tip and lowest at the basal meristem (Laycock, 1974; Mazure and Field, 1980). Another study found that epiphytic bacterial communities on the kelp *Saccharina latissima* were more similar on older tissues from different individuals than between old and new (meristematic) tissues from the same individual (Staufenberger et al., 2008). The rapid growth of kelp blades provides new surface area for microbial colonization from the surrounding seawater, or other microbial reservoirs. There is some evidence that microbial symbionts on algae may be recruited from motile marine bacteria in the surrounding seawater. For example, motile marine bacteria exhibit chemotaxis toward phytoplankton cells and their extracellular products (Seymour et al., 2010, 2017). Extracted compounds from the brown algae *Fucus vesiculosus* mediated epiphytic microbial settlement and community composition onto artificial surfaces (Lachnit et al., 2013), and a recent kelp metagenome revealed a two- to threefold enrichment in flagellum, motility, and chemotaxis genes on the kelp surface relative to the surrounding seawater (Minich et al., 2018). Tracking microbial communities on meristemic vs. apical blade tissues over time can thus be used to examine how microbial communities assemble onto new kelp tissues and continue to develop on older tissues. To test whether tissue age affects microbial community assembly on the annual kelp *N. luetkeana*, we tracked microbial communities on new (meristematic) and old (apical) *N. luetkeana* blade tissues and the free-living microbial community in the surrounding seawater at one site throughout the spring and summer growing season.

## Materials and Methods

### Sample Collection

**FIGURE 1:**
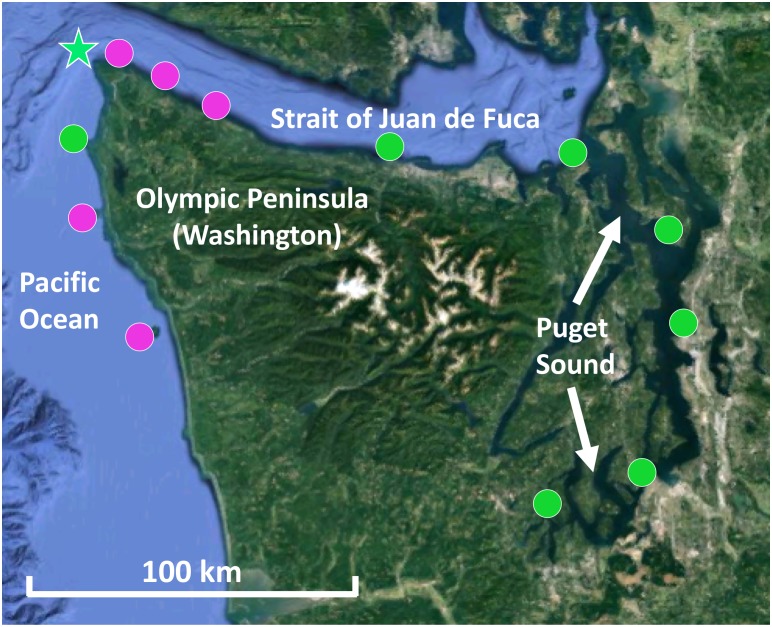
Map of kelp forests sampled in Washington, including co-occurring *N. luetkeana* and *M. pyrifera* kelp forests (pink circles) and locations where only *N. luetkeana* was sampled (green circles). The filled circles indicate kelp forests that were sampled for geographic comparisons (*n* = 12), while the green star indicates the location of Tatoosh Island, where temporal sampling was conducted.

Kelp blade tissue samples (*n* = 6 individuals) and seawater microbial samples (*n* = 2 or 3) were collected from 12 sites in Washington, including five sites on the outer Olympic Coast, three along the Strait of Juan de Fuca, and four within Puget Sound (Figure 1). For mixed kelp forests, both *N. luetkeana* and *M. pyrifera* tissues were collected. Kelp tissue samples were collected by boat from the floating kelp canopy by removing 2 × 1 cm^2^ of tissue from the middle of the kelp blade with sterile scissors. Individual samples were placed into 1.5 ml microcentrifuge tubes. Seawater samples were collected near the surface from within the kelp forest using sterile 1.0 L plastic bottles. After transporting seawater to the lab in a cooler with ice packs, microbial samples were collected by filtering seawater through 0.22 μm Millipore Sterivex^TM^ filters with a peristaltic pump. All kelp tissue and seawater samples were immediately frozen at -20°C until they were transferred to -80°C for storage until processing. Temperature and salinity were measured at each site using a Hydrolab MS5 water quality sonde (Bullman, Tatoosh, Koitlah) or a CastAway-CTD^®^ (all other sites).

Temporal sampling was conducted by monitoring one *N. luetkeana* population at the north-facing Main Beach site on Tatoosh Island (48.39°N, 124.74°W) at six time points from 12 May to 22 August 2017 (Figure 1). Kelp tissue samples were collected at low spring tides. At each time point, two tissue samples were collected from a single blade – one at the basal meristem, roughly 2 cm from where the blade connects to the stipe, to capture recently produced tissue (∼24–48 h old) and another near the apical end of the blade tip to sample older tissue (weeks to months old). Samples were collected from different kelp individuals (*n* = 6) at each time point. In contrast, samples from the 11 other sites across the geographic range were collected from the middle of the kelp blade (see above). *N. luetkeana* blade linear growth rates were measured during the time interval between each sampling date by putting a small 2 mm hole punch near the basal meristem of all censused kelp. Using this method to visually track growth rates near the base of the blade, we verified that meristem tissue collected at each census date was recently produced. In addition, the total length of each sampled blade was measured to determine the approximate age of apical (hereafter referred to as blade tip) samples.

### Molecular Methods

DNA was extracted from whole kelp tissues and seawater Sterivex^TM^ filters using the DNeasy Power Soil Kit (Qiagen). For seawater samples, Sterivex casings were cut with PVC cutters and half of the filter paper was removed and extracted as a solid sample (Jackrel et al., 2017). After DNA extraction, extracts were sent to Argonne National Laboratory for amplification of the V4 region of the 16S rRNA gene using the Earth Microbiome Project universal primers 515f–806r (Caporaso et al., 2011), with the modified forward primer (Parada et al., 2016) to reduce bias against *Crenarchaeota*, *Thaumarchaeota*, and the SAR11 clade (Walters et al., 2016). DNA amplicons were sequenced on an Illumina MiSeq paired-end run at Argonne National Laboratory following the procedures of Caporaso et al. (2012). Raw 16S rRNA sequences were deposited as FASTQ files to the Qiita platform^1^ (study ID #12016), through which all sequences and metadata were uploaded to the European Bioinformatics Institute (accession # PRJEB29319).

### 16S rRNA Sequence Data Processing and Statistical Analyses

Raw sequences were processed with QIIME2^2^. After raw sequence reads were demultiplexed, sequence quality control was performed, paired-end reads were merged, and amplicon sequence variants (ASVs) were generated using the Divisive Amplicon Denoising Algorithm (DADA2) (Callahan et al., 2016). With this algorithm, quality control included chimera detection and removal, sequence error elimination, singleton exclusion, and sequence trimming based on per-base-pair sequence quality graphs (both forward and reverse sequences were trimmed between 13 and 150 base pairs). The resulting sequences were classified with the Greengenes reference database (gg_13_8_99), trimmed to the V4 region. Note that the final bacterial order-level barplots were constructed using Silva taxonomy, because Green Genes taxonomy classifications did not go past the class level for many abundant ASVs (Supplementary Figure S1). After classification, chloroplast and mitochondria reads were removed. We used the “qiime diversity core-metrics-phylogenetic” function to analyze alpha and beta diversity among sample types, which requires rarefaction to a user-specified sampling depth prior to computing diversity metrics. For the geographic dataset (*n* = 115 total samples), we rarefied each sample to a depth of 1,000 sequences. For the temporal study of microbial community assembly on kelp blades, we found consistently low bacterial sequence abundances in kelp blade meristem samples; thus, we did not exclude low bacterial abundance meristem samples. Instead, for the temporal dataset (*n* = 80 total samples) we rarefied each sample to a depth of 200 sequences. To allow comparison between the geographic and temporal datasets, we used a sampling depth of 200 sequences per sample.

Alpha diversity indices (ASV richness, Shannon diversity, Faith’s phylogenetic diversity, and Pielou’s evenness) were calculated in QIIME2 with q2-diversity following the sequence depth normalizations listed above, and one-way non-parametric Kruskal–Wallis analyses of variance (ANOVA) were used to statistically compare the diversity indices for each sample type (*M. pyrifera* vs. *N. luetkeana* vs. seawater for geographic dataset, meristem vs. tip vs. seawater for temporal dataset). To compare overall microbial community structures between different sample types, one-way (fixed factor) non-parametric permutational multivariate ANOVA (PERMANOVA) were conducted based on unweighted unifrac distance matrices using q2-diversity beta-group-significance. Pairwise comparisons were conducted for significant PERMANOVA results with the Benjamini and Hochberg correction (*q*-value) to account for multiple pairwise comparisons. To ensure that differences in microbial community structure are not due to unequal dispersion of variability among groups, permutational multivariate analyses of dispersion (PERMDISP) were conducted for all significant PERMANOVA outcomes in PRIMER (version 6.1.11). Additional PERMANOVA tests were conducted in PRIMER test for (1) possible effects of geography on the distinction between microbial communities from each kelp species (two-way PERMANOVA with “species” as a fixed factor and “location” as a random factor, using only samples from locations where the two kelps co-occur) and (2) random effects of “kelp individual,” because meristem and tip communities were sampled from the same plant (two-way PERMANOVA with “meristem vs. tip” as a fixed factor and “kelp individual” as a random factor).

To search for differentially abundant microbial taxa across sample types or locations, analysis of composition of microbiomes (ANCOM) tests were implemented in QIIME2 (Mandal et al., 2015). To examine the variation in microbial communities that could be explained by environmental variables measured at each site (temperature and salinity), we performed a Constrained Analysis of Principal Coordinates (CAP) analysis (Anderson and Willis, 2003) based on Bray–Curtis dissimilarities in the R package Vegan (version 2.5-1; Oksanen et al., 2014). Statistical significance for CAP was determined with an ANOVA permutation test (anova.cca) using 1,000 permutations in Vegan. This constrained ordination was visually compared to an unconstrained ordination (PCoA) based on the same Bray–Curtis dissimilarity matrix. All statistical tests were examined with a post-correction experiment-wide error rate of 0.05. Differences in microbial communities were visualized in R (version 3.4.4) with the phyloseq package (McMurdie and Holmes, 2013) by creating (1) non-metric multidimensional scaling (NMDS) and principal coordinate analysis (PCoA) ordinations based on Bray–Curtis dissimilarities and (2) barplots and heatmaps based on relative abundances of microbial taxa across sample types.

### Phylogenetic Signal Methods

All phylogenetic structure analyses were implemented in R with the package “picante” (Kembel et al., 2010). Briefly, we created a phylogenetic distance matrix based on a maximum-likelihood 16S rRNA tree generated in QIIME2 with FastTree2 (Price et al., 2010). The net relatedness index (NRI) was calculated from the phylogenetic distance matrix using the “standardized effect size of pairwise distances in communities” function (ses.mpd) and the nearest taxa index (NTI) was calculated using the “standardized effect size of mean nearest taxon distances” function (ses.mntd). NRI is a measure of the mean relatedness between members of microbial communities, and the NTI is a measure of the smallest mean phylogenetic distance between all pairs of *n* taxa in a community sample. For both NRI and NTI analyses, the null model was randomized 999 times and set to “phylogeny.pool,” which randomly draws species from the phylogeny for its null distribution. NRI and NTI were calculated for microbial communities from each individual kelp tissue or seawater sample. Final graphs include only microbial community samples where *P* < 0.05. Positive NRI/NTI values describe a microbial community where members are on average more closely related to one another than they are to members of the randomized, regional (null model) microbial species pool. One-way ANOVA were run in R to statistically compare the NRI and NTI for each sample type (kelp meristem, kelp tip, seawater).

## Results

### Environmental Variables

Mean temperature increased with decreasing ocean influence, from a mean of 8.8°C at Destruction Island to 13.6°C at Squaxin Island, the location of the southernmost kelp bed in Puget Sound (Figure 1 and Supplementary Figure S2). Salinity showed the opposite pattern, as expected, decreasing with decreasing ocean influence from a mean of 33.45 at Destruction Island to 27.60 at Squaxin Island (Supplementary Figure S2). Note that Tatoosh Island displays the most variability (Supplementary Figure S2), because while most locations were only sampled once on the day that microbial samples were collected, temperature and salinity data were collected at Tatoosh Island multiple times throughout the summer, from June until August. Despite this variability, there is a strong pattern of increasing temperature and decreasing salinity as you move across the 13 locations, from the Pacific Ocean to the inner Puget Sound.

### Comparative Analysis of *M. pyrifera* and *N. luetkeana* Microbial Communities

**FIGURE 2:**
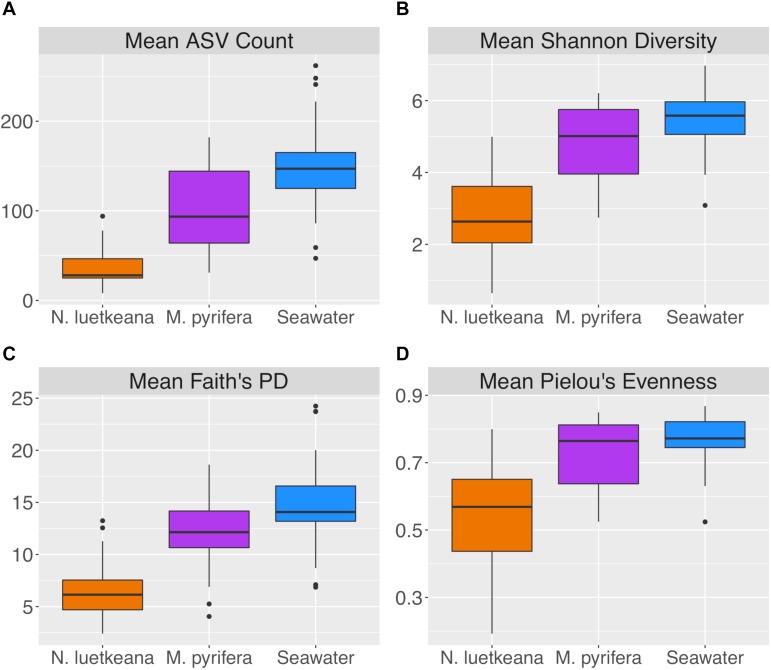
Alpha diversity indices including **(A)** mean ASV count, **(B)** mean Shannon diversity, **(C)** mean Faith’s phylogenetic diversity, and **(D)** mean Pielou’s evenness for *N. luetkeana*, *M. pyrifera*, and seawater microbial communities.

After removing samples with less than 1,000 sequences, the spatial dataset contained *n* = 28 *M. pyrifera* samples from 5 sites, *n* = 59 *N. luetkeana* samples from 11 sites, and *n* = 28 seawater samples from 9 sites. *N. luetkeana* samples from one location (Bullman Beach) had consistently low sequence abundances and were excluded from all analyses. Across all sites, alpha diversity metrics including mean ASV species richness, Shannon diversity, Faith’s phylogenetic diversity, and Pielou’s Evenness were significantly higher on *M. pyrifera* than *N. luetkeana*, and all alpha diversity metrics were significantly higher in seawater compared to both kelp species (Kruskal–Wallis ANOVA, *df* = 2; *H* = 82.98 and *P* < 0.001 for ASV richness, *H* = 75.81 and *P* < 0.001 for Shannon diversity, *H* = 74.64 and *P* < 0.001 for Faith’s PD, *H* = 61.32 and *P* < 0.001 for Pielou’s Evenness; corrected *P*-values < 0.001 for all Kruskal–Wallis pairwise tests; Figure 2). Mean ASV richness per sample was 36 for *N. luetkeana* blades, 102 for *M. pyrifera* blades, and 147 for seawater microbial communities.

**FIGURE 3:**
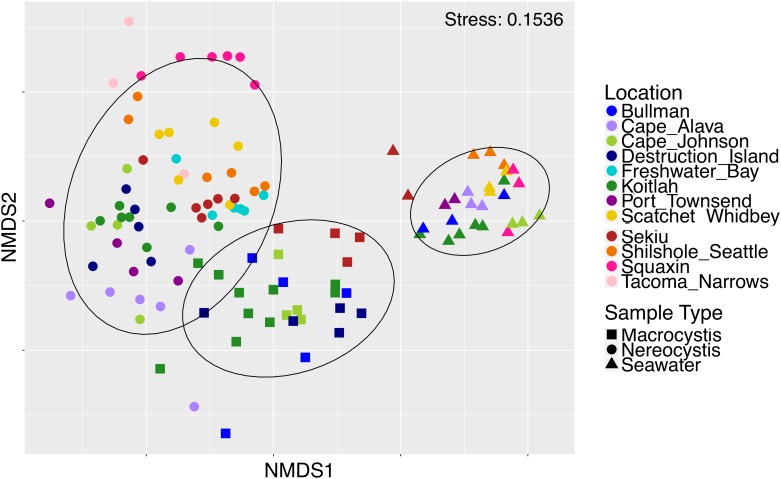
Non-metric multidimensional scaling plot of *M. pyrifera*, *N. luetkeana*, and seawater microbial communities across 12 sites in Washington.

Epiphytic microbial communities hosted by the canopy-forming kelps *N. luetkeana* and *M. pyrifera* were distinct from each other and from the surrounding seawater, despite co-occurrence of these two species in mixed kelp forests (Table 1A; all PERMANOVA pairwise comparisons *q*-value = 0.001; Figure 3). Because *N. luetkeana* were sampled at sites where *M. pyrifera* did not occur, we eliminated the potential that geography confounded differences between the species by testing for the effect of species only at the four sites where they do co-occur (Supplementary Table S1). This restricted PERMANOVA demonstrated that *N. luetkeana* and *M. pyrifera* still hosted significantly different microbial communities at these sites (Supplementary Table S1). Although the PERMDISP test was significant (Table 1A), indicating unequal dispersion of variability among groups, the significance resulted from a difference in dispersion between each kelp species and seawater (pairwise test for *N. luetkeana* and seawater: *t* = 6.67, *P* = 0.001; pairwise test for *M. pyrifera* and seawater: *t* = 3.40, *P* = 0.004), while *N. luetkeana* and *M. pyrifera* microbial communities did not display significant differences in dispersion (PERMDISP pairwise: *t* = 1.78, *P* = 0.112). *N. luetkeana* and *M. pyrifera* hosted significantly different microbial communities (PERMANOVA pairwise, *df* = 1, pseudo-*F* = 9.84, *q*-value = 0.001), yet the relative abundances of the top bacterial phyla, classes, and orders on *N. luetkeana* and *M. pyrifera* were quite similar (Table 2). The top bacterial phyla on each kelp were *Proteobacteria* (74.9% for *N. luetkeana*; 76.5% for *M. pyrifera*), followed by *Bacteroidetes* (11.8% for *N. luetkeana*; 16% for *M. pyrifera*), *Verrucomicrobia* (10.5% for *N. luetkeana*; 1.3% for *M. pyrifera*), and *Planctomycetes* (2.1% for *N. luetkeana*; 3.9% for *M. pyrifera*). These four phyla accounted for 98–99% of all microbial taxa on each kelp species. The top classes on *N. luetkeana* and *M. pyrifera* included *Gammaproteobacteria* (49 and 47%, respectively), *Alphaproteobacteria* (25 and 28%), *Verrucomicrobiae* (11 and 1%), *Saprospirae* (9 and 10%), *Flavobacteriia* (3 and 6%), and *Planctomycetia* (2 and 3%; Table 2). While bacteria made up most of the microbial communities on kelp, we detected one archeal phylum hosted by *M. pyrifera* (*Crenarchaeota*, <0.001% mean relative abundance) and one by *N. luetkeana* (*Euryarchaeota*, <0.001% mean relative abundance).

**Table 1:** Results of statistical tests comparing beta diversity (variation in microbial community structure) among **(A)** kelp species and geographic locations, and **(B)** kelp meristem and tip vs. date.

	PERMANOVA			PERMDISP		
(A) Geographic groups compared	*df*	Pseudo-*F*	*P*	*df*	*F*	*P*
*Macrocystis*, *Nereocystis*, seawater	2	18.78	0.001^∗^	2	23.57	0.001^∗^
*Macrocystis* by 5 locations	4	1.98	0.001^∗^	4	3.34	0.141
*Nereocystis* by 11 locations	10	2.40	0.001^∗^	10	1.63	0.429
Seawater by 9 locations	8	2.16	0.001^∗^	8	10.96	0.012^∗^
**(B) Temporal groups compared**						
Meristem, tip, seawater	2	4.00	0.001^∗^	2	3.89	0.043^∗^
Meristem by date	5	1.44	0.002^∗^	5	1.58	0.479
Tip by date	5	3.57	0.001^∗^	5	3.69	0.102
Seawater by date	4	2.09	0.001^∗^	4	6.78	0.226


*For all significant PERMANOVA results, PERDISP tests were conducted to ensure that significant results were not due to unequal dispersion of variability among groups*.

**Table 2:** Mean relative abundances (proportion out of 1.0 ± SE) of top bacterial classes and mean relative abundances of orders on *M. pyrifera* (*n* = 28) and *N. luetkeana* (*n* = 59), across all locations.

Phylum	Class	*M. pyrifera* mean abundance (±SE)	*N. luetkeana* mean abundance (±SE)	*M. pyrifera* orders (mean abundance)	*N. luetkeana* orders (mean abundance)
Proteobacteria	Gammaproteobacteria	0.47 (±0.03)	0.49 (±0.03)	Chromatiales (0.05), Thiohalorhabdales (0.04), Alteromonadales (0.03), Thiotrichales (0.03), Legionellales (0.01)	Chromatiales (0.06), Thiohalorhabdales (<0.01), Alteromonadales (0.02), Thiotrichales (<0.01), Legionellales (<0.01)
Proteobacteria	Alphaproteobacteria	0.28 (±0.02)	0.25 (±0.03)	Rhodobacterales (0.24), Rhizobiales (0.01), Sphingomonadales (0.01), BD7-3 (0.01), Rickettsiales (0.01)	Rhodobacterales (0.24), Rhizobiales (>0.01), Sphingomonadales (>0.01), BD7-3 (>0.01), Rickettsiales (>0.01)
Verrucomicrobia	Verrucomicrobiae	0.01 (±0.01)	0.11 (±0.01)	Verrucomicrobiales (0.01)	Verrucomicrobiales (0.11)
Bacteroidetes	Saprospirae	0.10 (±0.02)	0.09 (±0.02)	Saprospirales (0.10)	Saprospirales (0.09)
Bacteroidetes	Flavobacteriia	0.06 (±0.01)	0.03 (±0.01)	Flavobacteriales (0.06)	Flavobacteriales (0.03)
Planctomycetes	Planctomycetia	0.03 (±0.01)	0.02 (±0.00)	Pirellulales (0.03)	Pirellulales (0.02)
Proteobacteria	Deltaproteobacteria	0.01 (±0.00)	0.01 (±0.00)	Bdellovibrionales (0.01)	Bdellovibrionales (0.01)
Actinobacteria	Acidimicrobiia	0.01 (±0.00)	<0.01 (±0.00)	Acidimicrobiales (0.01)	Acidimicrobiales (<0.01)
	Unknown	0.02 (±0.00)	<0.01 (±0.00)	Unknown (0.33)	Unknown (0.41)


*Table includes all bacterial classes and orders that have a mean abundance >0.01 for each species*.

At the ASV level, *N. luetkeana* and *M. pyrifera* microbial communities were more distinct. Across all sites, *N. luetkeana* hosted 1,046 unique ASVs from 21 bacterial and 1 archaeal phyla and *M. pyrifera* hosted 1,643 ASVs from 23 bacterial and 1 archaeal phyla. *N. luetkeana* and *M. pyrifera* shared 33 and 21% of unique microbial ASVs, respectively. Interestingly, only 32% of kelp ASVs on each species were also found in the surrounding seawater (334 out of 1,046 ASVs for *N. luetkeana* and 527 out of 1,643 for *M. pyrifera*). Of the top 10 most abundant ASVs on *N. luetkeana* and *M. pyrifera*, many were shared and four had high relative abundances on both kelps (Table 3). The single most abundant ASV on *M. pyrifera*, *Granulosicoccus* sp. (*Gammaproteobacteria*), was the third most abundant ASV on *N. luetkeana*, making up about 17% of the total microbial communities on both kelps (Table 3). The top two ASVs on *N. luetkeana*, another *Granulosicoccus* sp. (22% of total community) and an *Alphaproteobacteria* from the family *Hyphomonadaceae* (19%), were present at very low abundance on *M. pyrifera* (<1%; Table 3).

**Table 3:** Mean relative abundances (proportion out of 1.0 ± SE) and taxonomic information for the 10 most abundant ASVs on *M. pyrifera* and *N. luetkeana*, from all sampling locations.

*M. pyrifera* mean abundance	*N. luetkeana* mean abundance	Seawater mean abundance	Phylum	Class	Order	Family
**0.178 (±0.02)**	**0.173 (±0.03)**	**<0.001**	**Proteobacteria**	**Gamma-proteobacteria**	**Chromatiales**	**Granulosicoccaceae**
**0.075 (±0.01)**	**0.015 (±0.00)**	**<0.001**	**Proteobacteria**	**Alpha-proteobacteria**	**Rhodobacterales**	**Hyphomonadaceae**
0.059 (±0.02)	<0.001	<0.001	Proteobacteria	Gamma- proteobacteria	Chromatiales	Granulosicoccaceae
**0.039 (±0.01)**	**0.057 (±0.01)**	**<0.001**	**Proteobacteria**	**Gamma-proteobacteria**	**Chromatiales**	**Granulosicoccaceae**
0.033 (±0.00)	0.011 (±0.00)	<0.001	Proteobacteria	Alpha-proteobacteria	Rhodobacterales	Hyphomonadaceae
0.029 (±0.01)	<0.001	<0.001	Proteobacteria	Gamma-proteobacteria	Chromatiales	Granulosicoccaceae
0.018 (±0.02)	0.002 (±0.00)	<0.001	Proteobacteria	Gamma-proteobacteria	Thiohalorhabdales	Unknown
0.016 (±0.00)	<0.001	<0.001	Proteobacteria	Alpha-proteobacteria	Rhodobacterales	Hyphomonadaceae
**0.015 (±0.00)**	**0.016 (±0.00)**	**<0.001**	**Planctomycetes**	**Planctomycetia**	**Pirellulales**	**Pirellulaceae**
0.015 (±0.00)	0.003 (±0.00)	0.003	Proteobacteria	Alpha-proteobacteria	Rhodobacterales	Hyphomonadaceae
0.009 (±0.00)	0.186 (±0.03)	<0.001	Proteobacteria	Alpha-proteobacteria	Rhodobacterales	Hyphomonadaceae
0.009 (±0.00)	0.220 (±0.03)	<0.001	Proteobacteria	Gamma-proteobacteria	Chromatiales	Granulosicoccaceae
0.003 (±0.00)	0.014 (±0.00)	<0.001	Bacteroidetes	Saprospirae	Saprospirales	Saprospiraceae
0.001 (±0.00)	0.030 (±0.01)	0	Bacteroidetes	Saprospirae	Saprospirales	Saprospiraceae
<0.001	0.027 (±0.01)	<0.001	Bacteroidetes	Saprospirae	Saprospirales	Saprospiraceae
<0.001	0.050 (±0.01)	0.001	Verrucomicrobia	Verrucomicrobiae	Verrucomicrobiales	Verrucomicrobiaceae


*Mean abundances of each ASV in surrounding seawater samples are included. The four bolded ASVs have a high mean abundance on both kelp species*.

### Geographic Variation in Kelp Blade Microbial Communities

**FIGURE 4:**
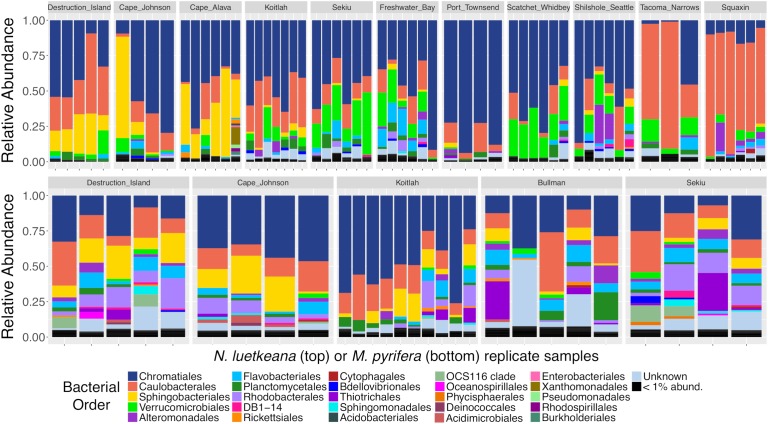
Relative abundance barplots of kelp blade microbial communities from *N. luetkeana* (top row) and *M. pyrifera* (bottom row) across 11 and 5 sites, respectively. Colors represent bacterial orders classified by Silva taxonomy. Sites are listed from left to right in order of decreasing ocean influence, from the outer coast (Destruction Island, Cape Johnson, Cape Alava, and Koitlah) to the Strait of Juan de Fuca (Sekiu, Freshwater Bay, and Port Townsend) and Puget Sound (Scatchet Whidbey, Shilshole Seattle, Tacoma Narrows, and Squaxin Island).

Both *N. luetkeana* and *M. pyrifera* microbial communities displayed significant spatial variation across 11 (*N. luetkeana*) and 5 (*M. pyrifera*) geographically separated kelp forests (Table 1A and Figure 4). Further, PERMANOVA tests were significant for each species vs. location while PERMDISP tests were not, indicating that significant differences in beta diversity are not due to differences in dispersion (Table 1A). *M. pyrifera* displayed significantly different microbial communities at Sekiu compared to the other four sites, and Koitlah was different from three out of four other sites (Supplementary Table S2). Differential abundance analysis with ANCOM revealed four ASVs with significantly different relative abundances across *M. pyrifera* sites (Supplementary Table S3). Three ASVs had a higher relative abundance on *M. pyrifera* from Sekiu compared to the other four sites, and they were classified as: (1) family *Francisellaceae* (*Gammaproteobacteria*), (2) *Granulosicoccus* sp. (*Gammaproteobacteria*), and (3) family *Saprospiraceae* (*Bacteroidetes*). The fourth differentially abundant ASV, also classified as *Granulosicoccus* sp. (*Gammaproteobacteria*), was absent at Sekiu but present at the other four sites.

Across 11 sites, *N. luetkeana* displayed significant spatial variation in microbial community composition (Supplementary Table S2 and Figure 4). Notably, the sites at either end of the geographic sampling, Cape Alava and Squaxin, had significantly different microbial communities from all other sites (PERMANOVA pairwise comparisons, *q*-value < 0.05, Supplementary Table S2). Sites closer in proximity tended to have more similar microbial communities, and a gradient of microbial community composition spans the 11 sites sampled (Figure 4). CAP analysis revealed that while salinity was significantly correlated with microbial community composition in the CAP model (*F* = 1.90, *df* = 1, *P* = 0.035), temperature was not (*F* = 1.22, *df* = 1, *P* = 0.284). However, temperature and salinity together only accounted for 5% of the variation in microbial communities across the geographic gradient (Supplementary Figure S3). In the unconstrained (PCoA) ordination, the first two axes explained 44.4% of the variation in microbial community structure (Supplementary Figure S3B), while the first two axes of the CAP ordination constrained by temperature and salinity only explained 0.053% of the variation in the same dataset (Supplementary Figure S3A), further indicating that measured environmental variables were not important predictors of microbial community structure.

Analysis of composition of microbiomes detected that 31 ASVs contributed to the spatial variation in *N. luetkeana* microbial communities across the 11 sites (Supplementary Table S3). Of these, ASVs from the family *Saprospiraceae* (*Bacteroidetes*) had a much higher abundance on *N. luetkeana* from the outer coast of Washington compared to sites in the Strait of Juan de Fuca and Puget Sound (yellow bars corresponding to order *Sphingobacteriales* in Figure 4 and Supplementary Table S3). The third most abundant bacterium on *N. luetkeana*, classified as *Granulosicoccus* sp. (*Gammaproteobacteria*), was significantly less abundant at the Southern Puget Sound sites (Tacoma Narrows and Squaxin) than on the outer coast and in the Strait of Juan de Fuca (navy blue bars corresponding to order *Chromatiales* in Figure 4 and Supplementary Table S3). While it was not detected as significantly different with ANCOM, one *Alphaproteobacteria* (*Robiginitomaculum* sp. family *Hyphomonadaceae*) had a much greater relative abundance in the Southern Puget Sound sites. This ASV had a mean abundance of 10–37% of all sequences at outer coast sites, 1–9% in the Strait of Juan de Fuca, and it increased to 50–77% of all sequences at Tacoma Narrows and Squaxin Island, the two Southern Puget Sound sites (pink bars in Figure 4). This was also the second most abundant ASV on *N. luetkeana* across all sites, with a mean relative abundance of 18.6% (Table 3). Microbes that had altered abundances across the geographic gradient were not differentially abundant in the surrounding seawater at these sites (Supplementary Figure S4). Other notable differentially abundant bacteria included ASVs belonging to the families *Flavobacteriaceae* (*Bacteroidetes*) and *Verrucomicrobiaceae* (*Verrucomicrobia*), which were both more abundant on *N. luetkeana* from the Strait of Juan de Fuca and the upper Puget Sound than on the outer coast or in Southern Puget Sound (Supplementary Table S3).

### Comparative Analysis of Kelp and Seawater Microbial Communities

Microbial communities in the surrounding seawater contained 3,725 unique ASVs from 42 bacterial and 3 archaeal phyla. Seawater microbial communities were dominated by the phyla *Bacteroidetes* (50%), *Proteobacteria* (42%), *Verrucomicrobia* (4%), and *Actinobacteria* (2%). Archeal phyla in the seawater included *Crenarchaeota* (0.12% mean relative abundance), *Euryarchaeota* (0.01%), and *Parvarchaeota* (<0.001%). At the class level, seawater contained a high abundance of *Flavobacteriia* (48%), followed by *Alphaproteobacteria* (25%), *Gammaproteobacteria* (15%), *Verrucomicrobiae* (4%), and *Betaproteobacteria* (1%). Of the 3,725 unique seawater ASVs, 334 of them were also found on *N. luetkeana* and 527 were found on *M. pyrifera*. All but one of the top 10 most abundant ASVs found on each kelp species were also found in the surrounding seawater, but at extremely low abundances of <0.03% (Table 3). Kelp forest seawater microbial communities displayed significant spatial variation (Table 1A and Supplementary Table S4), but significant PERMDISP tests also indicated differences in dispersion (Table 1A). A greater percentage of pairwise site comparisons were significantly different for *N. luetkeana* and *M. pyrifera* blade microbial communities (69 and 60%, respectively) than for seawater microbial communities (29%; Supplementary Tables S1, S3), suggesting that the kelp microbiome has greater spatial variation than surrounding seawater microbial communities. This difference in kelp vs. seawater spatial variation is also visible on the NMDS plot (Figure 3).

### Microbial Abundance and Diversity on New and Old *N. luetkeana* Blade Tissues

**FIGURE 5:**
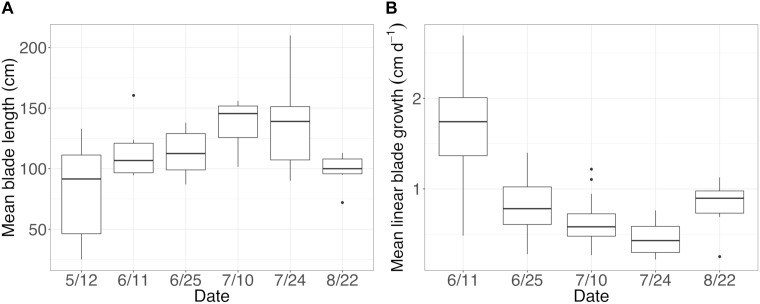
**(A)** Mean (±standard error) length of sampled blades and **(B)** mean (±standard error) linear blade growth rates throughout the growing season.

On Tatoosh Island, *N. luetkeana* linear blade growth rates were measured in intervals from mid-May to late August, averaging 0.84 cm per day. Blade growth rates were highest in early June (1.7 cm per day) and lowest in late July (0.45 cm per day, Figure 5). These growth rates demonstrate that basal blade meristem tissues were at most a few days old. In contrast, kelp blades increased in length throughout the growing season (Figure 5) and blade tip tissues were likely a few months old. After removing six meristem samples with <200 bacterial sequences, our dataset contained *n* = 30 *N. luetkeana* meristem samples (new tissue), *n* = 36 *N. luetkeana* blade tip samples (old tissue), and *n* = 14 seawater samples from six time points that spanned the growing season, from 12 May to 22 August. Across all dates, younger *N. luetkeana* meristem tissues hosted fewer bacterial sequences than samples from the older blade tips (paired *t*-test, *df* = 35, *t* = 16.16, *P* < 0.01), with an average of 961 bacterial sequences from the meristem and 39,661 bacterial sequences from the tip of the kelp blades (Supplementary Table S5). *N. luetkeana* meristem samples had a significantly higher abundance of chloroplast sequences than blade tips (paired *t*-test, *df* = 35, *t* = 6.36, *P* < 0.01), with an average of 24,212 chloroplast sequences from the meristem and 8,614 chloroplast sequences from the tip of the kelp blades (Supplementary Table S5). The meristem samples had very low percentage of bacterial sequences (1.4–7.4%) compared to chloroplast sequences (92.6–98.6%), while the older blade tip communities had a much higher percentage of bacterial sequences (67.4–90%) compared to chloroplast sequences (9.98–32.6%, Supplementary Table S5).

**FIGURE 6:**
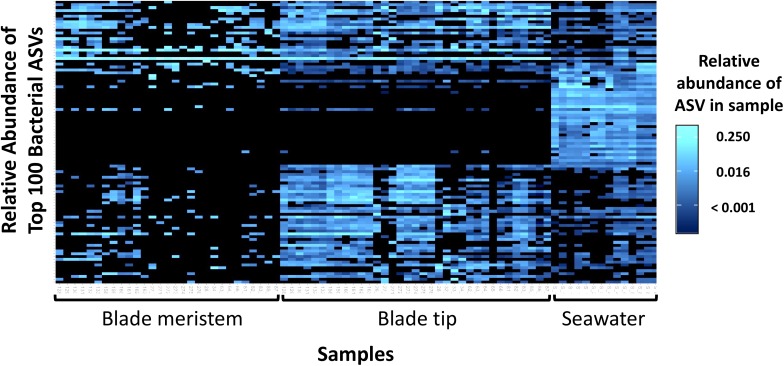
Heatmap showing the relative abundances of the 100 most abundant bacterial ASVs from *N. luetkeana* blade meristem, blade tip, and surrounding seawater microbial communities.

**FIGURE 7:**
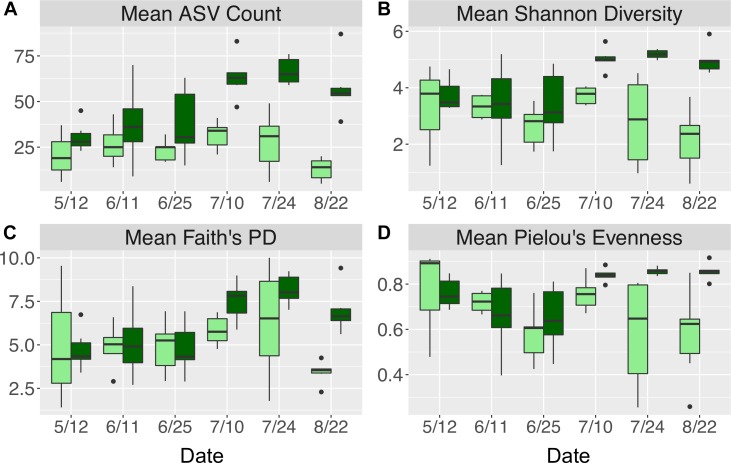
Alpha diversity indices including **(A)** mean (±standard error) ASV count, **(B)** mean (±standard error) Shannon diversity, **(C)** mean (±standard error) Faith’s phylogenetic diversity, and **(D)** mean (±standard error) Pielou’s evenness for *N. luetkeana* blade meristem (light green) and blade tip (dark green) microbial communities throughout the growing season.

Across all dates, alpha diversity metrics including mean ASV species richness, Shannon diversity, Faith’s phylogenetic diversity, and Pielou’s Evenness were significantly higher on blade tip than meristem microbial communities, but seawater communities had higher alpha diversity compared to kelp blades (Kruskal–Wallis ANOVA; *H* = 55.29 and *P* < 0.001 for ASV richness, *H* = 47.6 and *P* < 0.001 for Shannon diversity, *H* = 39.7 and *P* < 0.001 for Faith’s Phylogenetic Diversity, *H* = 30.98 and *P* < 0.001 for Pielou’s Evenness; corrected *P*-values < 0.03 for all Kruskal–Wallis pairwise tests). Mean ASV richness per sample was 49 for tip samples, 24 for meristem samples, and 81 for seawater microbial communities. Across all samples, the total observed diversity for meristem samples was 459 ASVs, while blade tip samples contained 1,408 ASVs. Of these microbial taxa, 201 ASVs were shared between *N. luetkeana* meristem and tip communities. Microbial communities in the surrounding seawater were the most diverse, with 3,115 total ASVs. The meristem communities shared 42% of ASVs with the surrounding seawater microbial community (191 total shared ASVs), while only 14% (201 ASVs) of blade tip ASVs were found in the seawater. Most of the ASVs with the highest relative abundance on *N. luetkeana* blades were also found in the surrounding seawater microbial communities, but many of the most abundant seawater ASVs were absent from the *N. luetkeana* blade microbial community (Figure 6). Throughout the summer, alpha diversity indices generally increased in blade tip microbial communities, reaching a peak in July and August, while ASV count and Shannon Diversity in meristem communities remained low (Figure 7). Interestingly, the gradual increase and end of July maximum in blade tip ASV count, Shannon Diversity, and Faith’s Phylogenetic Diversity matched the trend observed in the length of the sampled blades, where mean blade length increased throughout the summer and peaked in July, decreasing slightly in August (Figure 5).

### Microbial Community Composition and Successional Dynamics on New and Old *N. luetkeana* Blade Tissues

**FIGURE 8:**
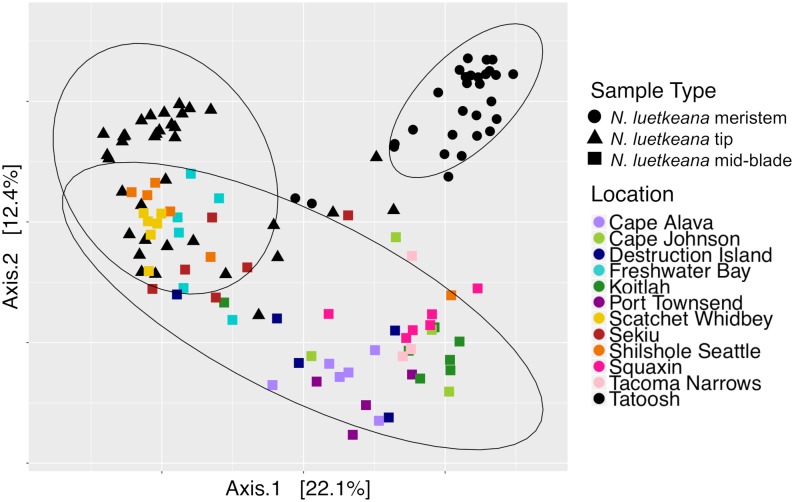
Principal coordinate analysis (PCoA) plot of all *N. luetkeana* microbial communities across 12 sites in Washington, combining the *N. luetkeana* mid-blade samples from the geographic sampling (squares) with the temporal dataset from Tatoosh Island, WA (black circles = *N. luetkeana* blade meristem, black triangles = *N. luetkeana* blade tip).

Across all time points, microbial communities on *N. luetkeana* tissue from the meristem (new tissue) and tip of the blade (old tissue) were significantly different from one another and from the surrounding seawater (Table 1B; meristem–tip pairwise, *df* = 1, pseudo-*F* = 6.29, *q*-value = 0.004; Figure 8). There was no effect of kelp individual, as a random factor, on this difference between meristem and tip microbial communities (Supplementary Table S1). While the PERMDISP test was significant (Table 1B), the significance resulted from a difference in dispersion between the kelp and seawater (pairwise test for tip vs. seawater: *t* = 2.32, *P* = 0.063; pairwise test for meristem vs. seawater: *t* = 2.95, *P* = 0.012), while *N. luetkeana* meristem and tip microbial communities did not display significant differences in dispersion (PERMDISP pairwise: *t* = 0.703, *P* = 0.516). Many of the most abundant bacterial ASVs on the *N. luetkeana* blade tip tissues were present on the blade meristem, but at much lower relative abundances (Figure 6). *N. luetkeana* blade tip microbial communities displayed significant temporal variation (Table 1B). Each date was distinct (PERMANOVA pairwise comparisons, *q*-value < 0.05), except for 11 June and 25 June (PERMANOVA pairwise, *df* = 1, pseudo-*F* = 1.46, *q*-value = 0.163). *N. luetkeana* meristem communities also displayed significant temporal variation (Table 1B). However, of the six dates, the only significant difference was between meristem microbial communities from 10 July and 22 August (PERMANOVA pairwise comparison, *df* = 1, pseudo-*F* = 2.57, *q*-value = 0.045). While the overall test for seawater microbial communities indicated temporal variation (Table 1B), none of the dates were significantly different from one another after correction for multiple pairwise tests (PERMANOVA pairwise comparisons, *q*-value > 0.133).

**FIGURE 9:**
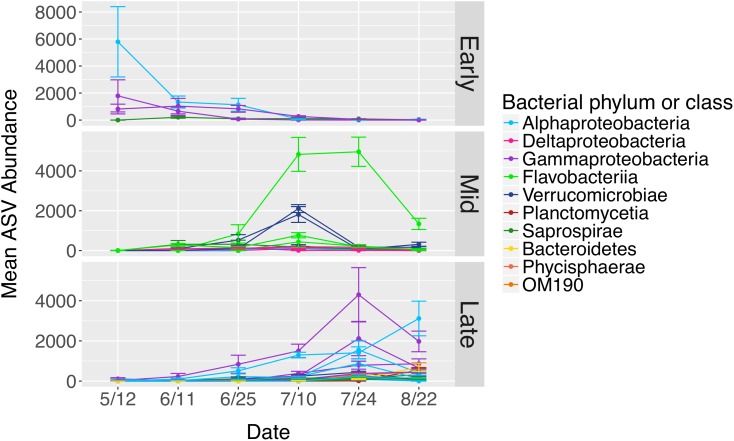
Mean abundances of ASVs (*n* = 40) from *N. luetkeana* blade tip microbial communities that displayed significantly different abundances across time with ANCOM. The ASVs that displayed significant temporal patterns were classified as early, mid, and late successional taxa based on when they were most abundant in blade tip communities. Each ASV is plotted individually and colored by bacterial class.

The total number of bacterial sequences was invariant across time on blade tip samples (ANOVA, *df* = 5, *F* = 1.87, *P* = 0.131), allowing us to identify differentially abundant taxa over time. *N. luetkeana* blade tip samples contained 40 microbial ASVs that significantly differed in relative abundance through time (ANCOM, *W*-scores range from 1249 to 1385). The ASVs that displayed significant temporal patterns using ANCOM were classified as early, mid, and late successional taxa based on when they were most abundant in blade tip communities. Early successional microbes included bacteria from the classes *Alphaproteobacteria*, *Gammaproteobacteria* (*Granulosicoccus* sp.), and *Saprospirae* (Figure 9). In particular, one *Alphaproteobacteria* (family *Hyphomonadaceae*) ASV decreased from a mean abundance of 5,794 sequences per samples in May down to six sequences per sample in late July (Figure 9). Mid-successional ASVs included *Flavobacteriia* (family *Flavobacteriaceae*, *Olleya* sp., and *Tenacibaculum* sp.), *Verrucomicrobiae*, *Alphaproteobacteria*, *Gammaproteobacteria* (*Vibrio* sp.), *Deltaproteobacteria* (order *Bdellovibrionales*), and *Saprospirae* (Figure 9). Of the mid-successional ASVs, one *Flavobacteriia* (family *Flavobacteriaceae*) increased from a mean abundance of 0 in May up to 4,827 and 4,960 in early and late July, decreasing back to 1,339 in late August. Two *Verrucomicrobiae* (*Roseibacillus* sp.) ASVs increased from a mean abundance of 0 in May up to 1,811 and 2,109 in early July, decreasing back to abundances of 315 and 166 in late August (Figure 9). Late-successional ASVs were the most numerous and included members of the class *Gammaproteobacteria* (*Granulosicoccus* sp. and the order *Thiohalorhabdales*), *Alphaproteobacteria* (*Octadecabacter antarcticus* and families *Rickettsiaceae*, *Hyphomonadaceae*, and *Erythrobacteraceae*), *Phycisphaerae*, *Planctomycetia*, *Saprospirae*, *Verrucomicrobiae*, *Flavobacteriia*, and *OM190* (Figure 9). Late-successional ASVs that were particularly abundant included one *Gammaproteobacteria* (*Granulosicoccus* sp.) ASV that increased from a mean abundance of 28 in May up to 4,296 in late July, and one *Alphaproteobacteria* (family *Hyphomonadaceae*) ASV that increased from a mean abundance of 7 in May up to 3,117 in late August (Figure 9).

The total number of bacterial sequences on meristem samples was significantly different over time (ANOVA, *df* = 5, *F* = 4.77, *P* = 0.003; Supplementary Table S5). In contrast to tip communities, ANCOM only revealed five ASVs in *N. luetkeana* meristem communities that differed in relative abundance through time (ANCOM, *W*-scores range from 485 to 538). These included one *Gammaproteobacteria* (family *HTCC2089*), one *Bacteroidetes* (*Aquimarina* sp.), and three ASVs from the class *Verrucomicrobiae* (*Rubritalea* sp.).

### Phylogenetic Signal in Microbial Community Assembly

**FIGURE 10:**
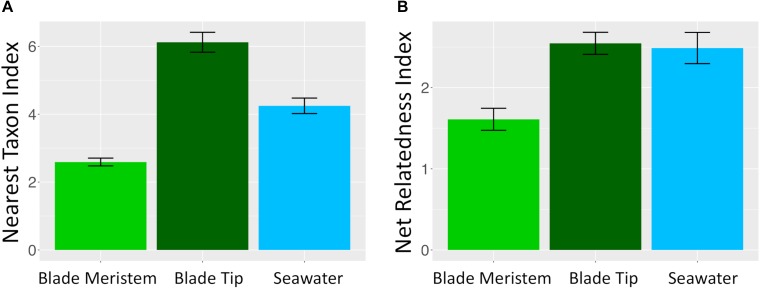
Mean (±standard error) **(A)** nearest taxon index and **(B)** NRI of microbial communities on *N. luetkeana* blade meristem (*n* = 30), *N. luetkeana* blade tip (*n* = 36), and surrounding seawater samples (*n* = 22) from Tatoosh Island, WA.

Microbial taxa from kelp and seawater communities displayed significant phylogenetic clustering (Figure 10). Members of kelp and surrounding seawater communities were more closely related to one another than would be expected by chance if microbial communities were randomly assembled from a regional species pool of all detected kelp and seawater microbial taxa. For the NTI, all *n* = 36 *N. luetkeana* blade tip samples, all *n* = 22 seawater samples, and *n* = 18 out of 30 *N. luetkeana* meristem samples displayed significant phylogenetic clustering (*P* < 0.05; Figure 10). For the NRI, *n* = 26 out of 36 *N. luetkeana* blade tip samples, *n* = 13 out of 22 seawater samples, and *n* = 9 out of 30 *N. luetkeana* meristem samples displayed significant phylogenetic clustering (*P* < 0.05; Figure 10). The NTI was positive for all samples that displayed significant phylogenetic signal, but differed significantly among kelp meristem, kelp tip, and seawater samples (ANOVA, *df* = 2, *F* = 42.9, *P* < 0.001). For NTI, all three groups were significantly different from one another – kelp meristem communities displayed the lowest NTI, followed by seawater, and kelp tip microbial communities had the highest NTI values (Tukey HSD *post hoc* pairwise tests, *P* < 0.001; Figure 10). The NRI also differed significantly among kelp meristem, kelp tip, and seawater samples (ANOVA, *df* = 2, *F* = 7.29, *P* < 0.01), with a significantly lower NRI from kelp meristem microbial communities than kelp tip or seawater communities (Tukey HSD *post hoc* pairwise tests, *P* < 0.01; Figure 10). The NRI of kelp tip and seawater microbial communities did not differ (Tukey HSD *post hoc* pairwise tests, *P* = 0.96).

### Comparing Geographic and Temporal Variation in *N. luetkeana* Microbial Communities

Across all samples from both spatial and temporal datasets, the *N. luetkeana* meristem (*n* = 30), blade tip (*n* = 36), and mid-blade microbial communities from the geographic dataset (*n* = 59) were significantly different (PERMANOVA, *df* = 2, pseudo-*F* = 7.55, *P* = 0.001; all pairwise comparisons *q*-value = 0.001). This is visible on the PCoA plot of combined spatial and temporal datasets, where microbial communities from the three sample types form distinct clusters (Figure 8). The total number of ASVs found on *N. luetkeana* increased from blade meristem (459 ASVs) to mid-blade samples (1,046 ASVs) and was highest in blade tip communities (1,408 ASVs), despite a higher number of samples from mid-blade microbial communities. Kelp tissues from Tatoosh Island (*N. luetkeana* meristem and tip samples from all dates) were significantly different from *N. luetkeana* mid-blade microbial communities collected at the 11 other sites (PERMANOVA, *df* = 11, pseudo-*F* = 2.53, *P* = 0.001; all pairwise comparisons against Tatoosh Island, *q*-value < 0.04). While we only tracked temporal variation at Tatoosh, the temporal variation there only overlapped partially with the large amount of spatial variation that we observed across the other 11 sites (Figure 8), suggesting that geographic differences in kelp blade microbial communities and local successional dynamics are distinct.

## Discussion

### Microbial Community Structure and Composition on Canopy-Forming Kelps

The two canopy forming kelp in the northeast Pacific host distinct microbial communities on their blades, consistent with previous studies reporting host-specific microbial communities associated with macroalgae (Lachnit et al., 2009; Aires et al., 2016; Lemay et al., 2018). *N. luetkeana* and *M. pyrifera* shared only 33 and 21% of unique microbial ASVs, respectively. Similarly, Lemay et al. (2018) found that 37% of bacterial taxa were shared among eight kelp species. However, a recent meta-analysis of macroalgal microbial communities found that many families of epiphytic bacteria are generalists commonly found on red, green, and brown algal hosts (Florez et al., 2017), suggesting that common properties of the macroalgae microbiome may promote the growth of similar microbial taxa. Many of the abundant kelp-associated bacterial families that we detected (*Rhodobacteraceae*, *Flavobacteriaceae*, *Alteromonadaceae*, *Saprospiraceae*) were found to be generalist macroalgal associates by Florez et al. (2017). We found that relative abundances of the top bacterial phyla, classes, and orders on *N. luetkeana* and *M. pyrifera* were remarkably similar. Microbial communities on each species were dominated by *Proteobacteria* (∼75% of all sequences), followed by *Bacteroidetes*, *Verrucomicrobia*, and *Planctomycetes*. The only large phylum-level difference in microbial communities between the two kelps was *Verrucomicrobia*; this phylum made up 10% of microbial sequences on *N. luetkeana*, and only 1% on *M. pyrifera*. Despite similar compositions at higher microbial taxonomic levels, blades of *N. luetkeana* and *M. pyrifera* each host a unique microbiome at the ASV level.

Relatively few extremely abundant ASVs dominated the microbial symbiont community of *N. luetkeana*, while *M. pyrifera* hosted a more diverse community with fewer dominant symbionts. Supporting this observation, Pielou’s evenness was significantly greater for *M. pyrifera* than *N. luetkeana* microbial communities. The three most abundant ASVs on *N. luetkeana* averaged 58% of the total microbial symbiont community, while the combined abundances of the top 20 symbionts made up the same proportion of the *M. pyrifera* microbial community. The most abundant ASV on *N. luetkeana* (mean abundance 22%) was *Granulosicoccus* sp. (*Gammaproteobacteria*). Bacteria from the genus *Granulosicoccus* have been isolated from seagrass (Kurilenko et al., 2010) and brown algae (Park et al., 2014), and they have been characterized as aerobic, chemoheterotrophic bacteria that are capable of nitrate reduction to nitrite (Baek et al., 2014; Park et al., 2014). Three of the four most abundant ASVs on *N. luetkeana* were classified as *Granulosicoccus* sp., indicating that there are multiple, closely related sequence variants that dominate the microbiome of *N. luetkeana.* The second most abundant ASV on *N. luetkeana*, an *Alphaproteobacteria* from the family *Hyphomonadaceae* (mean abundance 19%), displayed a large amount of spatial variation across sites. It was highly abundant on the outer coast (10–37%), had a very low abundance in the Strait of Juan de Fuca (1–9%), and became very dominant in Puget Sound, making up 50–77% of all sequences from kelp at the two Southern Puget Sound sites. This ASV was further classified as *Robiginitomaculum* sp. A bacterium in this genus, isolated from Antarctic seawater, was characterized as obligately aerobic, chemoheterotrophic, and capable of nitrate reduction (Lee et al., 2007). While these two *N. luetkeana* symbionts were present at <1% relative abundance on *M. pyrifera*, the single most abundant ASV on *M. pyrifera*, *Granulosicoccus* sp., was the third most abundant ASV on *N. luetkeana* and made up 17% of microbial communities on both kelps. The second most abundant ASV on *M. pyrifera* (mean abundance 8%), an *Alphaproteobacteria* from the family *Hyphomonadaceae*, was present at lower abundances (1.5%) on *N. luetkeana.*

We found that across all sites, the perennial kelp *M. pyrifera* hosted a more diverse microbiome than the annual kelp *N. luetkeana*. *M. pyrifera* had a mean ASV richness per sample of 102 and hosted a total of 1,643 unique ASVs, while *N. luetkeana* had a mean richness of 36 ASVs per sample and hosted 1,046 unique ASVs. Faith’s phylogenetic diversity was significantly greater on *M. pyrifera* compared to *N. luetkeana*, suggesting that the increase in the number of microbial taxa found on *M. pyrifera* is due to associations with many microbial lineages, rather than association with taxa from one or a few particular taxonomic groups. Our data support the hypothesis that thallus longevity allows perennial kelps to accumulate more mature and highly colonized microbial communities compared with annuals (Lemay et al., 2018). Further, it has been hypothesized that perennial tissue could provide a reservoir of kelp-associated microbes for the colonization of new tissues (Bengtsson et al., 2010; Lemay et al., 2018). We found that *N. luetkeana* shared a similar proportion (33%) of its microbial community with the perennial *M. pyrifera* as with the surrounding seawater (32%), suggesting that both the surrounding seawater and perennial kelps may contribute to microbial community assembly on annual kelp blades. While the microbiome of each kelp species was clearly distinct from the seawater microbial community, both canopy-forming kelps shared a remarkably similar proportion of microbes with the seawater (32%). The fraction of kelp-associated microbial taxa detected in the surrounding seawater is reported to be highly variable across studies, from as low as 2% (Michelou et al., 2013) to as high as 86% (Lemay et al., 2018), and may reflect a broad array of differences among studies, including differences in sequencing depth or differences in retention time of seawater in proximity to the kelp.

### Geographic Variation in Kelp Blade Microbial Communities

Blades of *N. luetkeana* and *M. pyrifera* exhibited significant variation in microbial communities across 11 (*N. luetkeana*) and 5 (*M. pyrifera*) geographically distinct kelp forests. Other studies have reported regional differences in microbial symbiont composition associated with eight kelp species located <100 km apart (Lemay et al., 2018), as well as the kelp *Ecklonia radiata* from different habitat types located <10 km apart (Marzinelli et al., 2018) and >1,000 km apart (Marzinelli et al., 2015). In our study, *N. luetkeana* forests were located 10–400 km apart, but experience very different environmental conditions (Supplementary Figure S2). Kelp forests in Puget Sound are exposed to warmer temperatures and lower nutrient concentrations, while outer coast kelp experience cooler temperatures and higher nutrient concentrations due to coastal upwelling. Additionally, Southern Puget Sound kelp forests experience greater anthropogenic stressors than outer coast kelp forests (Pfister et al., 2018). Elevated temperatures led to a disruption in the blade surface microbiome of *M. pyrifera* (Minich et al., 2018). Despite a range of 9–14°C, we found that salinity but not temperature was significantly correlated with microbial community composition on *N. luetkeana* blades. However, both environmental variables together only accounted for 5% of the variation in *N. luetkeana* microbial communities across the spatial gradient, suggesting that these environmental variables are not an important determinant of microbial community composition in our study. However, we note that the temperature and salinity values only represent a snapshot in time and a subset of the many abiotic factors influencing these sites.

Our analysis revealed regional differences in the relative abundance of certain microbial taxa across the spatial gradient. In particular, ASVs from the family *Saprospiraceae* (*Bacteroidetes*) had higher abundances on the outer coast, while Southern Puget Sound sites displayed a relative increase in the family *Hyphomonadaceae* (*Alphaproteobacteria*) and a decrease in the relative abundance of *Granulosicoccus* sp. (*Gammaproteobacteria*). *Saprospiraceae* are among the core microbial symbionts associated with the red algae *Porphyra umbilicalis* (Miranda et al., 2013) and they were found on *N. luetkeana* meristem communities in Vancouver, Canada (Chen and Parfrey, 2018). This group of microbes may play a role in metabolizing complex carbon resources (McIlroy and Nielsen, 2014). Microbes that had altered abundances across the geographic gradient were not differentially abundant in the surrounding seawater at these sites, suggesting that the kelp blades surfaces may be highly selective environments that promote the growth of certain microbial taxa under different conditions.

### Temporal Variation, Microbial Community Succession, and Phylogenetic Signal

Our temporal study demonstrated that microbial communities on the meristem of *N. luetkeana* blades have much lower species diversity, evenness, and likely lower total microbial abundances than the older and more developed microbial communities on the tip of the kelp blade. Another study found that *Laminaria saccharina* meristem tissue hosted less diverse microbial communities than older thallus tissue (Staufenberger et al., 2008). Because *N. luetkeana* blades grew linearly ∼1 cm per day (Figure 5), meristem tissues hosted microbial communities that were at most a few days old. Kelp blades gradually increased in length throughout our study, thus blade tip microbial communities were likely a few months old (Figure 5). Many water column bacteria are motile (Fenchel, 2001), and microbial metagenomes on kelp contained a high abundance of motility genes (Minich et al., 2018), suggesting that microbes may be recruited from the surrounding seawater onto the kelp surface. Meristem microbial communities were more similar to the surrounding seawater (42% ASVs shared with seawater) than the kelp blade tip communities (14% shared ASVs), suggesting possible recruitment of seawater microbes onto new kelp tissues. However, it is also possible that meristem communities contained a larger proportion of seawater microbes due to the small number of bacterial sequences on kelp meristems (Supplementary Table S5), and possible contamination from seawater.

While kelp meristem microbial communities were almost temporally stable, with a difference between only two of the six dates, we found significant temporal variation in blade tip microbial communities across all dates. On the kelp blade tip, we found evidence for microbial community succession, including increasing ASV richness and evenness through time and thus kelp age. Interestingly, ASV richness, Shannon diversity, and Pielou’s Evenness on blade tip communities reached a maximum by early July, remaining high but not increasing further through the end of August (Figure 7). Other studies have reported similar patterns of temporal variation, including an increase in microbial species richness on the brown algae *C. compressa* over its annual growth cycle (Mancuso et al., 2016) and an increase in microbial richness and evenness with tissue age on the kelp *L. hyperborea* (Bengtsson et al., 2012). The 40 ASVs that showed significantly different relative abundances on blade tip microbial communities across time also displayed clear patterns of early, mid, and late successional taxa (Figure 9). Interestingly, one of the abundant mid-successional taxa, *Olleya* sp. (*Flavobacteriaceae*), may produce exopolysaccharides (Nichols, 2005). Given these patterns of microbial community succession, we hypothesize that the addition of microbial taxa to the kelp surface biofilm facilitates the recruitment of additional microbial taxa by providing potentially novel metabolic substrates and increasing microbial niche space (Rivett et al., 2016).

Interestingly, we found that phylogenetic similarity among taxa in the microbiome was higher in blade tip communities than in meristem communities. Microbial communities in both kelp and seawater samples were more phylogenetically clustered than expected by chance, a pattern that has been reported for seawater microbial communities (Pontarp et al., 2012). Both the number and phylogenetic relatedness (NTI and NRI indices) of microbial taxa increased with kelp tissue age, suggesting that microbes were added to the kelp surface microbial community deterministically. Phylogenetic clustering may be consistent with kelp acting as selective filters, promoting the growth of specific microbial taxa that might fill similar niche space in the kelp surface microbiome. It may also indicate that interactions among microbes are repeatedly important in the colonization process that occurs throughout the kelp growing season.

## Conclusion

Canopy-forming kelps create vast and highly productive underwater forests in temperate marine ecosystems (Wheeler and Druehl, 1986), yet we are just beginning to gain perspective on the importance of the microbial symbiont communities to these foundational species. Here, we found evidence for host-specificity and geographic variation in microbial communities on the blades of *M. pyrifera* and *N. luetkeana*, two canopy-forming kelps with different life histories. We demonstrated temporal variation in microbial communities over the summer growing season on blades of the annual kelp *N. luetkeana*. By tracking both geographic and temporal variation in kelp microbial communities, we confirm previous studies showing that one or the other factor is important (Staufenberger et al., 2008; Bengtsson et al., 2010; Marzinelli et al., 2015; Mancuso et al., 2016), revealing that both processes simultaneously shape microbial symbiont communities on kelps. Our temporal study demonstrated that microbial communities on younger, meristematic blade tissue were less developed, displaying significantly lower ASV diversity and evenness than communities at the tip of the blade. Further, we found that relatedness among microbial taxa increased from basal to apical blade tissues, suggesting that the process of microbial community assembly on kelp blades may be deterministic. To understand why certain groups of closely related microbes are abundant on kelp blades, future research should focus on identifying the functions of the dominant symbionts to determine their importance to the host kelp.

## Author Contributions

BW and CP designed the experiments and contributed edits and revisions. BW performed the field sampling and analyzed the data. BW wrote the first draft of the manuscript.

## Conflict of Interest Statement

The authors declare that the research was conducted in the absence of any commercial or financial relationships that could be construed as a potential conflict of interest.

## Footnotes

1 https://qiita.ucsd.edu/

2 https://qiime2.org
