# Supplementary material for: Successional Dynamics and Seascape-Level Patterns of Microbial Communities on the Canopy-Forming Kelps Nereocystis luetkeana and Macrocystis pyrifera
Source: Front Microbiol. 2019 Feb 26;10:346. doi: 10.3389/fmicb.2019.00346 (PMC6399156; doi:10.3389/fmicb.2019.00346)
Supplement: Supplementary file 1 [file Data_Sheet_1.PDF]

Supplementary Material

Figure S1. Barplots showing variation in the relative abundances of bacterial orders in kelp blade microbial communities from *N. luetkeana* (top row) and *M. pyrifera* (bottom row) across 11 and 5 sites, respectively. Colors represent bacterial orders classified by Green Genes taxonomy. Sites are in order of decreasing ocean influence, from the outer coast (Destruction Island, Cape Johnson, Cape Alava and Koitlah) to the Strait of Juan de Fuca (Sekiu, Freshwater Bay, and Port Townsend) and Puget Sound (Scatchet Whidbey, Shilshole Seattle, Tacoma Narrows, and Squaxin Island).

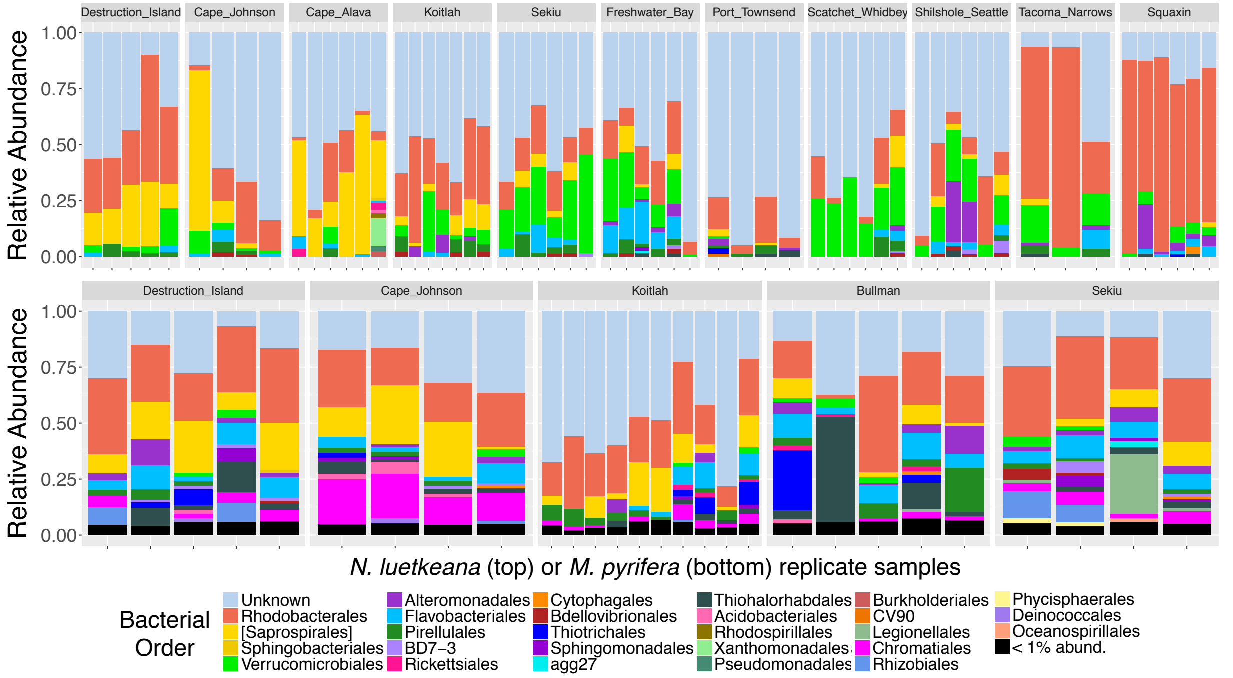

Figure S2. Mean (± standard error) temperature and salinity at sites across the geographic gradient. Sites are in order of increasing ocean influence, from Squaxin (inner Puget Sound) to Destruction Island (outer coast Washington on the Pacific Ocean).

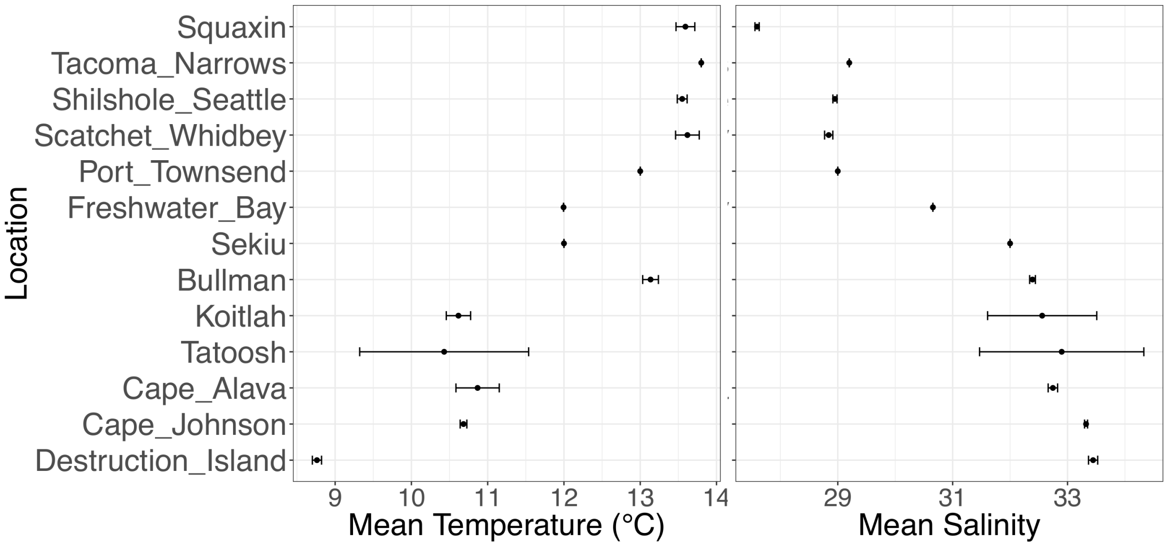

Figure S3. Ordination analysis of sequence data using **A)** Constrained Analysis of Principal Coordinates (CAP) and **B)** unconstrained Principal Coordinates Analysis (PCoA) based on Bray-Curtis distances for *N. luetkeana* microbial communities from different sites across the geographic gradient (colored circles, same for both plots). For the constrained (CAP) plot, microbial communities are shown in relation to temperature and salinity.

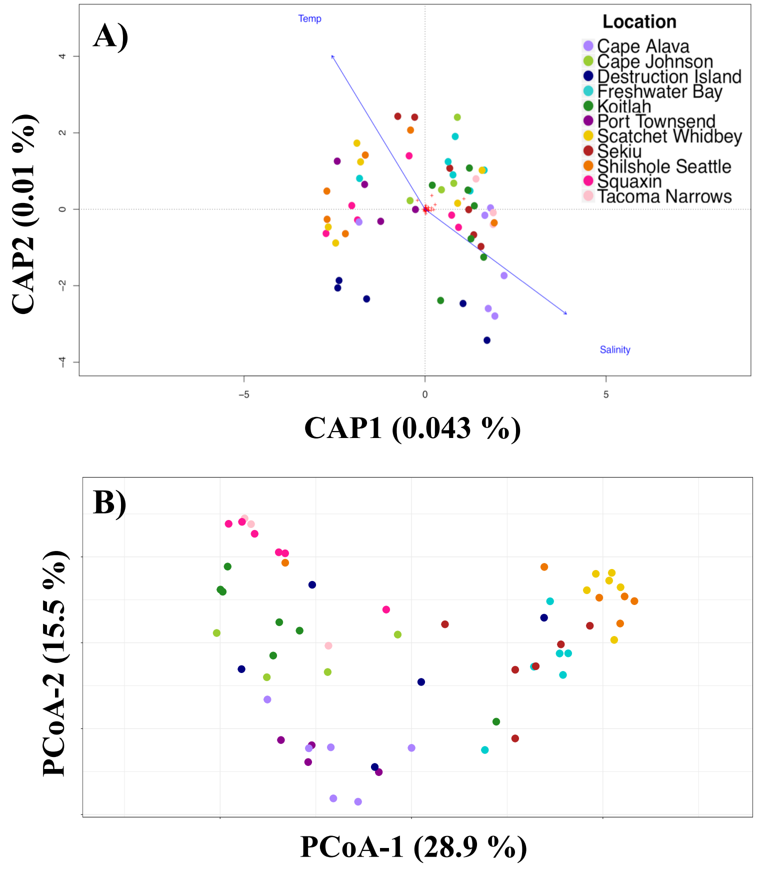

Figure S4. Barplots showing variation in the relative abundances of the bacterial families *Hyphomonadaceae* (class *Alphaproteobacteria*; top row) and *Saprospiraceae* (phylum *Bacteroidetes*; bottom row) in *N. luetkeana* blade microbial communities (left side) and seawater microbial communities (right side) across sites. Sites are in order of decreasing ocean influence, from the outer coast (Destruction Island, Cape Johnson, Cape Alava and Koitlah) to the Strait of Juan de Fuca (Sekiu, Freshwater Bay, and Port Townsend) and Puget Sound (Scatchet Whidbey, Shilshole Seattle, Tacoma Narrows, and Squaxin Island).

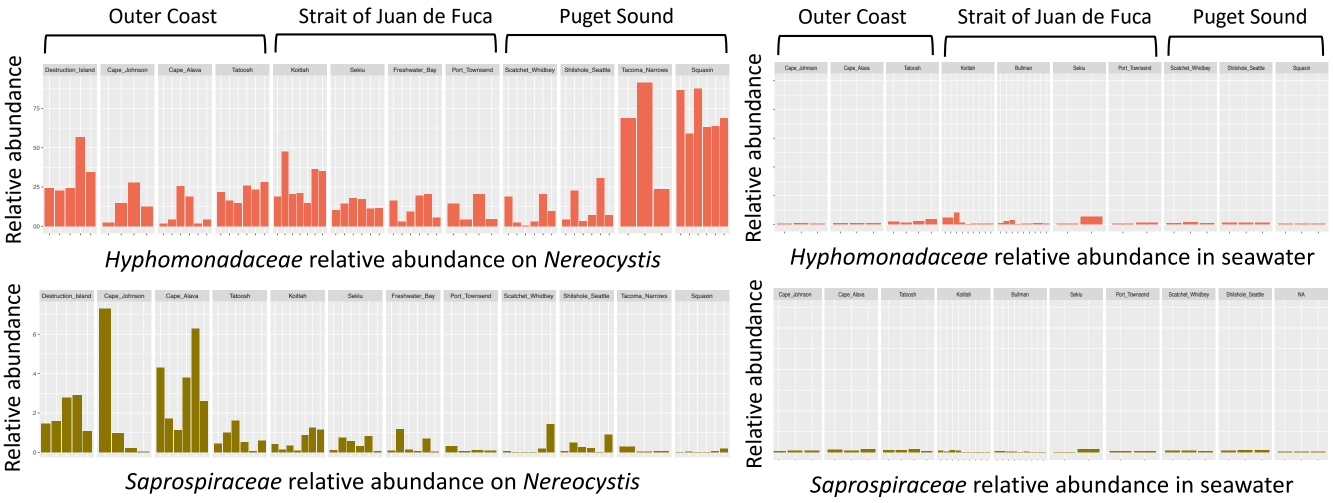

Table S1. Additional results of beta diversity tests comparing microbial communities among a) the two kelp species only at geographic locations where they co-occur (two-way PERMANOVA with "species" as a fixed and "location" as a random factor), and b) kelp meristem vs. tip, while controlling for kelp individual (two-way PERMANOVA with "meristem vs. tip" as a fixed factor and "kelp individual" as a random factor).

		PERMANOVA	
**a) Effect of kelp species only at co-occurring locations**	**df**	**pseudo-F (or *t*)**	**P**
Location (random, 4 levels)	3	F = 3.03	0.001*
Species (fixed, 2 levels)	1	F = 4.74	0.015*
N. luetkeana – M. pyrifera pairwise		*t* = 2.17	0.012*
Species*Location	3	F = 2.58	0.001*
N. luetkeana – M. pyrifera pairwise @ Sekiu		*t* = 2.27	0.007*
N. luetkeana – M. pyrifera pairwise @ Koitlah		*t* = 2.66	0.001*
N. luetkeana – M. pyrifera pairwise @ Cape Johnson		*t* = 2.26	0.028*
N. luetkeana – M. pyrifera pairwise @ Destruction Is.		*t* = 1.85	0.005*
**b) Meristem – tip analysis**			
Meristem vs. tip (fixed, 2 levels)	1	F = 25.49	0.001*
Effect of "kelp individual" (random, 36 levels)	35	F = 1.12	0.182

Table S2. PERMANOVA pairwise comparisons of kelp microbial communities at each location for the overall tests of *N. luetkeana* vs. location (11 sites) and *M. pyrifera* vs. location (5 sites).

Location 1	Location 2	Species	pseudo-F	p-value	q-value
**Destruction_Island**	Cape_Johnson	*N. luetkeana*	1.72	0.06	0.08
**Destruction_Island**	Cape_Alava	*N. luetkeana*	2.72	0.00	**0.01**
**Destruction_Island**	Koitlah	*N. luetkeana*	1.34	0.19	0.20
**Destruction_Island**	Sekiu	*N. luetkeana*	3.15	0.00	**0.01**
**Destruction_Island**	Freshwater_Bay	*N. luetkeana*	5.09	0.01	**0.02**
**Destruction_Island**	Port_Townsend	*N. luetkeana*	1.25	0.22	0.23
**Destruction_Island**	Scatchet_Whidbey	*N. luetkeana*	2.72	0.00	**0.01**
**Destruction_Island**	Shilshole_Seattle	*N. luetkeana*	3.21	0.01	**0.03**
**Destruction_Island**	Tacoma_Narrows	*N. luetkeana*	2.99	0.02	**0.03**
**Destruction_Island**	Squaxin	*N. luetkeana*	4.36	0.01	**0.02**
**Cape_Johnson**	Cape_Alava	*N. luetkeana*	1.88	0.01	**0.02**
**Cape_Johnson**	Koitlah	*N. luetkeana*	1.01	0.43	0.43
**Cape_Johnson**	Sekiu	*N. luetkeana*	1.34	0.11	0.13
**Cape_Johnson**	Freshwater_Bay	*N. luetkeana*	2.21	0.03	**0.04**
**Cape_Johnson**	Port_Townsend	*N. luetkeana*	1.39	0.09	0.11
**Cape_Johnson**	Scatchet_Whidbey	*N. luetkeana*	1.21	0.14	0.15
**Cape_Johnson**	Shilshole_Seattle	*N. luetkeana*	1.52	0.07	0.09
**Cape_Johnson**	Tacoma_Narrows	*N. luetkeana*	1.42	0.06	0.08
**Cape_Johnson**	Squaxin	*N. luetkeana*	1.99	0.01	**0.02**
**Cape_Alava**	Koitlah	*N. luetkeana*	2.75	0.00	**0.01**
**Cape_Alava**	Sekiu	*N. luetkeana*	3.72	0.00	**0.02**
**Cape_Alava**	Freshwater_Bay	*N. luetkeana*	4.71	0.01	**0.02**
**Cape_Alava**	Port_Townsend	*N. luetkeana*	2.09	0.02	**0.03**
**Cape_Alava**	Scatchet_Whidbey	*N. luetkeana*	2.81	0.00	**0.01**
**Cape_Alava**	Shilshole_Seattle	*N. luetkeana*	3.22	0.00	**0.01**
**Cape_Alava**	Tacoma_Narrows	*N. luetkeana*	2.69	0.01	**0.02**
**Cape_Alava**	Squaxin	*N. luetkeana*	3.53	0.00	**0.01**
**Koitlah**	Sekiu	*N. luetkeana*	1.70	0.04	**0.05**
**Koitlah**	Freshwater_Bay	*N. luetkeana*	3.37	0.01	**0.02**
**Koitlah**	Port_Townsend	*N. luetkeana*	1.56	0.10	0.12
**Koitlah**	Scatchet_Whidbey	*N. luetkeana*	1.79	0.01	**0.02**
**Koitlah**	Shilshole_Seattle	*N. luetkeana*	2.35	0.01	**0.02**
**Koitlah**	Tacoma_Narrows	*N. luetkeana*	2.12	0.01	**0.02**
**Koitlah**	Squaxin	*N. luetkeana*	3.77	0.00	**0.01**
**Sekiu**	Freshwater_Bay	*N. luetkeana*	1.44	0.12	0.13
**Sekiu**	Port_Townsend	*N. luetkeana*	3.20	0.01	**0.02**

**Sekiu**	Scatchet_Whidbey	*N. luetkeana*	1.63	0.02	**0.03**
**Sekiu**	Shilshole_Seattle	*N. luetkeana*	1.52	0.05	0.07
**Sekiu**	Tacoma_Narrows	*N. luetkeana*	1.87	0.01	**0.02**
**Sekiu**	Squaxin	*N. luetkeana*	3.49	0.00	**0.02**
**Freshwater_Bay**	Port_Townsend	*N. luetkeana*	4.10	0.01	**0.02**
**Freshwater_Bay**	Scatchet_Whidbey	*N. luetkeana*	2.41	0.02	**0.03**
**Freshwater_Bay**	Shilshole_Seattle	*N. luetkeana*	1.48	0.12	0.13
**Freshwater_Bay**	Tacoma_Narrows	*N. luetkeana*	2.06	0.02	**0.03**
**Freshwater_Bay**	Squaxin	*N. luetkeana*	3.08	0.01	**0.02**
**Port_Townsend**	Scatchet_Whidbey	*N. luetkeana*	2.13	0.01	**0.02**
**Port_Townsend**	Shilshole_Seattle	*N. luetkeana*	2.74	0.01	**0.02**
**Port_Townsend**	Tacoma_Narrows	*N. luetkeana*	2.23	0.06	0.08
**Port_Townsend**	Squaxin	*N. luetkeana*	3.08	0.00	**0.01**
**Scatchet_Whidbey**	Shilshole_Seattle	*N. luetkeana*	1.49	0.05	**0.07**
**Scatchet_Whidbey**	Tacoma_Narrows	*N. luetkeana*	1.28	0.12	0.13
**Scatchet_Whidbey**	Squaxin	*N. luetkeana*	2.23	0.00	**0.01**
**Shilshole_Seattle**	Tacoma_Narrows	*N. luetkeana*	1.41	0.11	0.13
**Shilshole_Seattle**	Squaxin	*N. luetkeana*	1.96	0.02	**0.03**
**Tacoma_Narrows**	Squaxin	*N. luetkeana*	1.69	0.02	**0.04**
**Destruction_Island**	Cape_Johnson	*M. pyrifera*	1.33	0.16	0.16
**Destruction_Island**	Koitlah	*M. pyrifera*	1.66	0.05	0.06
**Destruction_Island**	Bullman	*M. pyrifera*	1.50	0.04	0.06
**Destruction_Island**	Sekiu	*M. pyrifera*	2.46	0.01	**0.03**
**Cape_Johnson**	Koitlah	*M. pyrifera*	1.80	0.03	**0.05**
**Cape_Johnson**	Bullman	*M. pyrifera*	1.66	0.06	0.06
**Cape_Johnson**	Sekiu	*M. pyrifera*	2.98	0.03	**0.05**
**Koitlah**	Bullman	*M. pyrifera*	1.74	0.02	**0.05**
**Koitlah**	Sekiu	*M. pyrifera*	3.19	0.00	**0.02**
**Sekiu**	Bullman	*M. pyrifera*	1.87	0.03	**0.05**

Table S3. Differentially abundant ASVs detected with ANCOM on *N. luetkeana* (n = 31) and *M. pyrifera* (n = 4) across all sites. Each ASV is listed by taxonomic class, with lowest taxonomic classification indicated in parenthesis, along with the ANCOM W score. Mean relative abundances of ASVs across all sites are listed. Sites are listed left to right in order of decreasing ocean influence, from the outer coast (DSI = Destruction Island, CJO = Cape Johnson, CPA = Cape Alava and KOI = Koitlah) to the Strait of Juan de Fuca (BUL = Bullman, SEK = Sekiu, FWB = Freshwater Bay, and PTW = Port Townsend) and Puget Sound (SCW = Scatchet Whidbey, SHS = Shilshole Seattle, TAC = Tacoma Narrows, and SQX = Squaxin).

ASV	W score	Lowest Taxonomic Classification	DSI	CJO	CPA	KOI	SEK	FWB	PTW	SCW	SHS	TAC	SQX
**Nereo_1**	1045	Gammaproteobacteria (Granulosicoccus sp.)	2952	761	4839	877	1626	1898	4465	447	9	111	0
**Nereo_2**	1045	Bacteroidetes (Saprospiraceae)	930	152	1737	19	0	0	3	0	0	0	0
**Nereo_3**	1045	Bacteroidetes (Saprospiraceae)	1972	278	1240	11	0	0	21	1	0	0	0
**Nereo_4**	1018	Verrucomicrobia (Rubritalea sp.)	1257	25	227	137	2549	563	25	5459	387	35	60
**Nereo_5**	1007	Bacteroidetes (Flavobacteriaceae)	0	0	139	0	0	0	0	0	0	0	0
**Nereo_6**	1044	Bacteroidetes (Saprospiraceae)	476	0	96	4	0	0	18	5	0	0	0
**Nereo_7**	1044	Bacteroidetes (Saprospiraceae)	916	0	76	2	0	0	27	0	0	0	0
**Nereo_8**	1026	Bacteria (unclassified)	0	0	47	0	0	0	0	0	0	0	0
**Nereo_9**	1012	Alphaproteobacteria (Rickettsiales)	0	3	29	0	0	0	0	0	0	0	0
**Nereo_10**	998	Bacteroidetes (unclassified)	0	1	29	0	0	0	0	0	0	0	0
**Nereo_11**	996	Deltaproteobacteria (Bacteriovoracaceae)	15	11	15	0	0	0	0	0	0	0	0
**Nereo_12**	1027	Gammaproteobacteria (Pseudoalteromonas sp.)	0	5	5	0	0	5	1	46	35	1	1
**Nereo_13**	1033	Verrucomicrobia (Verrucomicrobiaceae)	15	7	1	170	364	131	0	366	1142	6	0
**Nereo_14**	966	Bacteroidetes (Flavobacteriaceae)	0	0	1	1	15	56	0	0	2	2	0
**Nereo_15**	1018	Verrucomicrobia (Verrucomicrobiaceae)	0	3	1	227	128	325	0	128	537	23	0
**Nereo_16**	1026	Alphaproteobacteria (Hyphomonadaceae)	0	6	0	0	161	176	0	209	233	0	0
**Nereo_17**	994	Alphaproteobacteria (Hyphomonadaceae)	0	0	0	100	157	172	0	0	72	4	0
**Nereo_18**	991	Alphaproteobacteria (Rickettsiales)	0	3	0	0	21	24	0	0	72	0	0
**Nereo_19**	1034	Bacteroidetes (Flavobacteriaceae)	17	0	0	2	236	554	0	224	370	2	0
**Nereo_20**	987	Bacteroidetes (Flavobacteriaceae)	0	0	0	0	0	147	0	21	11	0	0
**Nereo_21**	1029	Bacteroidetes (Saprospiraceae)	0	7	0	101	966	576	6	794	267	14	0

**Nereo_22**	992	Bacteroidetes (Saprospiraceae)	0	0	0	0	38	178	0	0	0	2	0
**Nereo_23**	1042	Gammaproteobacteria (unclassified)	0	0	0	113	18	0	0	0	0	0	0
**Nereo_24**	974	Gammaproteobacteria (Vibrio sp.)	0	0	0	0	0	0	0	34	14	0	0
**Nereo_25**	974	Planctomycetes (Class OM190)	0	0	0	31	14	79	0	0	0	0	0
**Nereo_26**	990	Planctomycetes (family Pirellulaceae)	0	0	0	0	94	379	0	17	11	16	0
**Nereo_27**	964	Verrucomicrobia (Persicirhabdus sp.)	11	11	0	65	248	1301	0	50	705	8	0
**Nereo_28**	1045	Gammaproteobacteria (Granulosicoccus sp.)	0	0	0	330	3474	3622	32	2159	1676	58	10
**Nereo_29**	993	Alphaproteobacteria (Hyphomonadaceae)	75	27	0	0	193	218	0	1298	232	3	12
**Nereo_30**	1034	Gammaproteobacteria (Granulosicoccus sp.)	0	0	0	0	75	185	0	6	41	0	12
**Nereo_31**	1030	Gammaproteobacteria (Thalassomonas sp.)	0	0	0	0	0	18	0	0	14	0	88

**ASV**	**W score**	**Lowest Taxonomic Classification**	**DSI**	**CJO**	**BUL**	**KOI**	**SEK**
**Macro_1**	1640	Gammaproteobacteria (Granulosicoccus sp.)	7	26	2	459	0
**Macro_2**	1555	Gammaproteobacteria (family Francisellaceae)	1	0	0	0	2377
**Macro_3**	1498	Gammaproteobacteria (Granulosicoccus sp.)	0	0	0	11	375
**Macro_4**	1543	Bacteroidetes (Saprospiraceae)	0	0	0	0	363

Table S4. PERMANOVA pairwise comparisons of seawater microbial communities at each location (9 sites).

Group 1	Group 2	pseudo-F	p-value	q-value
Cape_Johnson	Cape_Alava	2.53	0.11	0.12
Cape_Johnson	Koitlah	3.13	0.00	**0.04**
Cape_Johnson	Bullman	2.73	0.00	**0.04**
Cape_Johnson	Sekiu	2.00	0.10	0.12
Cape_Johnson	Port_Townsend	2.28	0.09	0.12
Cape_Johnson	Scatchet_Whidbey	2.79	0.10	0.12
Cape_Johnson	Shilshole_Seattle	3.51	0.11	0.12
Cape_Johnson	Squaxin	3.15	0.10	0.12
Cape_Alava	Koitlah	2.86	0.01	**0.04**
Cape_Alava	Bullman	2.68	0.01	**0.04**
Cape_Alava	Sekiu	2.01	0.11	0.12
Cape_Alava	Port_Townsend	2.27	0.11	0.12
Cape_Alava	Scatchet_Whidbey	2.54	0.11	0.12
Cape_Alava	Shilshole_Seattle	2.86	0.12	0.13
Cape_Alava	Squaxin	3.05	0.10	0.12
Koitlah	Bullman	1.32	0.06	0.12
Koitlah	Sekiu	1.88	0.02	0.07
Koitlah	Port_Townsend	1.47	0.02	0.06
Koitlah	Scatchet_Whidbey	1.56	0.01	**0.04**
Koitlah	Shilshole_Seattle	2.69	0.01	**0.04**
Koitlah	Squaxin	2.84	0.01	**0.04**
Bullman	Sekiu	1.58	0.06	0.12
Bullman	Port_Townsend	1.23	0.14	0.15
Bullman	Scatchet_Whidbey	1.62	0.01	**0.05**
Bullman	Shilshole_Seattle	2.60	0.00	**0.04**
Bullman	Squaxin	1.94	0.01	**0.04**
Sekiu	Port_Townsend	1.12	0.34	0.34
Sekiu	Scatchet_Whidbey	1.74	0.11	0.12
Sekiu	Shilshole_Seattle	1.69	0.10	0.12
Sekiu	Squaxin	1.94	0.09	0.12
Port_Townsend	Scatchet_Whidbey	1.89	0.09	0.12
Port_Townsend	Shilshole_Seattle	1.99	0.09	0.12
Port_Townsend	Squaxin	1.80	0.10	0.12
Scatchet_Whidbey	Shilshole_Seattle	1.79	0.10	0.12
Scatchet_Whidbey	Squaxin	2.57	0.10	0.12
Shilshole_Seattle	Squaxin	2.86	0.12	0.13

Table S5. Mean number of total 16S sequences, mean bacterial sequences and mean chloroplast sequences (as well as mean % chloroplast and bacterial sequences) for all *N. luetkeana*, *M. pyrifera*, and seawater samples.

Sample Type	Date	*n*	Total Sequences	Bacterial Sequences	Chloroplast Sequences	% Chloroplast	% Bacterial
***Nereocystis* meristem (Tatoosh)**	5/12/17	3	26,161	369	25,792	98.6	1.4
***Nereocystis* meristem (Tatoosh)**	6/11/17	4	26,178	419	25,759	98.4	1.6
***Nereocystis* meristem (Tatoosh)**	6/25/17	5	31,313	1,135	30,177	96.5	3.5
***Nereocystis* meristem (Tatoosh)**	7/10/17	6	31,811	1,392	30,420	95.8	4.2
***Nereocystis* meristem (Tatoosh)**	7/24/17	6	33,278	2,550	30,728	92.6	7.4
***Nereocystis* meristem (Tatoosh)**	8/22/17	6	22,057	320	21,738	98.6	1.4
***Nereocystis* tip (Tatoosh)**	5/12/17	6	42,967	30,132	12,834	32.6	67.4
***Nereocystis* tip (Tatoosh)**	6/11/17	6	45,636	35,547	10,090	25.7	74.3
***Nereocystis* tip (Tatoosh)**	6/25/17	6	46,808	39,140	7,667	17.7	82.3
***Nereocystis* tip (Tatoosh)**	7/10/17	6	54,983	49,532	5,451	10.0	90.0
***Nereocystis* tip (Tatoosh)**	7/24/17	6	52,888	47,500	5,388	11.1	88.9
***Nereocystis* tip (Tatoosh)**	8/22/17	6	45,667	35,014	10,653	27.3	72.7
**Seawater (Tatoosh)**	5/12/17	3	45,752	40,080	5,672	12	88
**Seawater (Tatoosh)**	6/11/17	2	42,812	35,431	7,382	18	82
**Seawater (Tatoosh)**	6/25/17	4	44,915	35,242	9,673	24	76
**Seawater (Tatoosh)**	7/24/17	2	49,269	44,231	5,038	10	90
**Seawater (Tatoosh)**	8/22/17	3	50,531	45,853	4,679	10	90
***Macrocystis* (Bullman)**	7/5/17	5	40,246	10,859	29,387	73.0	27.0
***Macrocystis* (Cape Johnson)**	7/31/17	4	38,907	27,549	11,358	26.5	73.5
***Macrocystis* (Destruction Island)**	8/1/17	5	40,726	29,344	11,382	32.1	67.9
***Macrocystis* (Koitlah)**	8/11/17	10	37,679	20,460	17,219	47.9	52.1
***Macrocystis* (Sekiu)**	7/2/17	4	45,463	30,834	14,629	32.5	67.5
***Nereocystis* (Cape Alava)**	8/2/17	6	35,700	13,643	22,057	62.0	38.0
***Nereocystis* (Cape Johnson)**	7/31/17	4	29,486	3,574	25,912	85.9	14.1
***Nereocystis* (Destruction Island)**	8/1/17	5	33,083	21,081	12,002	33.8	66.2
***Nereocystis* (Freshwater Bay)**	7/19/17	6	38,562	23,078	15,484	40.4	59.6
***Nereocystis* (Koitlah)**	8/11/17	7	40,969	6,402	34,567	85.8	14.2
***Nereocystis* (Port Townsend)**	7/19/17	4	29,185	9,642	19,543	67.9	32.1
***Nereocystis* (Scatchet Whidbey)**	8/10/17	6	47,728	30,977	16,751	35.4	64.6
***Nereocystis* (Sekiu)**	7/2/17	6	36,338	18,954	17,384	50.5	49.5
***Nereocystis* (Shilshole Seattle)**	8/10/17	6	46,159	21,258	24,901	56.1	43.9
***Nereocystis* (Squaxin)**	6/21/17	6	39,660	4,776	34,884	88.1	11.9
***Nereocystis* (Tacoma Narrows)**	8/19/17	3	29,472	2,150	27,323	92.4	7.6
**Seawater (Bullman)**	7/5/17	3	45,386	39,526	5,861	13.0	87.0
**Seawater (Cape Alava)**	8/2/17	3	53,451	49,665	3,786	7.4	92.6

**Seawater (Cape Johnson)**	7/31/17	3	39,032	22,628	16,404	43.4	56.6
**Seawater (Koitlah)**	8/11/17	6	40,398	27,924	12,474	31.6	68.4
**Seawater (Port Townsend)**	7/19/17	2	41,495	32,434	9,061	22.1	77.9
**Seawater (Scatchet Whidbey)**	8/10/17	3	71,919	67,661	4,258	8.5	91.5
**Seawater (Sekiu)**	7/2/17	2	41,297	29,226	12,072	30.9	69.1
**Seawater (Shilshole Seattle)**	8/10/17	3	96,088	93,957	2,131	2.2	97.8
**Seawater (Squaxin)**	6/21/17	3	50,251	45,674	4,578	9.3	90.7
